# Life tables of annual life expectancy and mortality for companion dogs in the United Kingdom

**DOI:** 10.1038/s41598-022-10341-6

**Published:** 2022-04-28

**Authors:** Kendy Tzu-yun Teng, Dave C. Brodbelt, Camilla Pegram, David B. Church, Dan G. O’Neill

**Affiliations:** 1 School of Veterinary Medicine, National Taiwan University, No. 1, Section 4, Roosevelt Rd, Da’an District, Taipei City, Taiwan; 2 Pathobiology and Population Sciences, The Royal Veterinary College, Hawkshead Lane, North Mymms, Hatfield, Herts AL9 7TA UK; 3 Clinical Science and Services, The Royal Veterinary College, Hawkshead Lane, North Mymms, Hatfield, Herts AL9 7TA UK

**Keywords:** Epidemiology, Ageing, Data processing

## Abstract

A life table is a tabulated expression of life expectancy and mortality-related information at specified ages in a given population. This study utilised VetCompass data to develop life tables for the UK companion dog population and broken down by sex, Kennel Club breed group, and common breeds. Among 30,563 dogs that died between 1st January 2016 and 31st July 2020, life expectancy at age 0 was 11.23 [95% confidence interval (CI): 11.19–11.27] years. Female dogs (11.41 years; 95% CI: 11.35–11.47) had a greater life expectancy than males (11.07 years; 95% CI: 11.01–11.13) at age 0. Life tables varied widely between breeds. Jack Russell Terrier (12.72 years; 95% CI: 12.53–12.90) and French Bulldog (4.53 years; 95% CI: 4.14–5.01) had the longest and shortest life expectancy at age 0, respectively. Life tables generated by the current study allow a deeper understanding of the varied life trajectory across many types of dogs and offer novel insights and applications to improve canine health and welfare. The current study helps promote further understanding of life expectancy, which will benefit pet owners and the veterinary profession, along with many other sectors.

## Introduction

A deeper understanding of life expectancies at different ages within the United Kingdom (UK) companion dog population, further categorised by sex and breed, is critical to the improvement of canine welfare and health management^1,2^. For example, existing and potential dog owners can develop realistic expectations for the typical remaining life period of their dogs through the knowledge of life expectancy. To date, much of the research on dog life expectancy has focused on reporting average overall ages at death in dogs that have been selected using referral or first opinion veterinary caseloads^1,3^, insurance databases^4^ or owner questionnaires^5,6^. Among companion dogs died between 2009 and 2011 in the UK, the median age at death was estimated to be 12.0 years [interquartile range (IQR): 8.9–14.2], and the median age at death for various breeds ranged from Dogue de Bordeaux at 5.5 years (IQR: 3.3–6.1; n = 21) to Miniature Poodle at 14.2 years (IQR: 11.1–15.6; n = 20)^1^.

Instead of offering a single value for the average age of at death, a life table is a tabulated expression of life expectancy and probability of death at different age groups of a given population. A life table provides much more detailed information and inference than a single summary average age at death across all ages^7^. There are two main types of life tables: (a) a *cohort life table*, which summarises the actual mortality experience of a group of individuals (the cohort) from the birth of the first to the death of the last member of the cohort, and (b) a *current life table*, which provides cross-sectional mortality and survival experience of a population during a single or few current years^8^. Cohort life tables have also been constructed using a hypothetical (i.e., not pre-determined) cohort^9,10^. Both types of life tables have their importance. Cohort life tables can inform the mortality situation of cohorts, but the data for current life tables are generally easier to be collected^8^.

Human life tables are routinely constructed for countries, or sub-populations within a country, as a proxy indicator of the general health of the population. A decrease in life expectancy implies that events leading to mortality occur, on average, earlier and is, therefore, suggestive of a generally less healthy population^11^. Thus, life tables can be used to monitor changes in the general health of a population over time, as well as to identify vulnerable (sub-)populations, promoting targeted investigation into the reasons for the observed reduction in life expectancy^11,12^. Human life tables are considered an essential tool for effective public planning and policy-making^13^, e.g., estimating the future costs of the Old-Age, Survivors, and Disability Insurance federal programmes within the United States^14^. In the UK, a national current life table for humans is generated every three years whereas there is an update every year in the United States^12,15^. National life tables are usually constructed for the population overall, per sex, and may include ethnic groups in some countries^16^.

Despite their usefulness for the management of human populations, life tables are rarely built for companion animals. Two life table studies for dogs were recently conducted in Japan^10,17^; the first created current life tables for dogs in general, along with estimates for differing sizes, using pet insurance data, whilst the other created a hypothetical cohort life table using pet cemetery data. These life tables have advanced the knowledge of dog life trajectory^18^ and have been applied in studies that required information on the life expectancy of dogs of different ages, such as a quantitative risk assessment of the introduction of rabies^19^ and the quantification of welfare impact caused by diseases^2^. However, given that the breed structure of dog populations can vary widely between countries, the international generalisability of lifetables needs to be considered carefully. In addition, the average lifespan and mortality profiles of individual breeds may differ among national dog populations for a wide range of genetic and healthcare reasons. For instance, on average, Labrador Retrievers lived 14.1 years (mean) in Japan^10^, 12.5 years (median) in the UK^1^, and 10.5 (median) years in Denmark^20^.

The construction of a life table for companion dogs in the UK could facilitate the understanding of the life expectancy and health of the UK companion dog population in a similar way to the application of such life tables in human populations^12,15^. A reliable canine life table can enhance our understanding of the life expectancy at different ages, as it demonstrates that life expectancy at each age is not the same as the average lifespan minus that age. There are practical implications when life expectancy is not understood correctly. For example, canine adoption centres may underestimate the typical remaining lifespan of adult dogs being rehomed if predictions about their age at death are based on their current age and the average lifespan. This could lead to a longer length of ownership than the adopting family had originally expected. Life tables for individual breeds could be particularly useful for informing decision-making for existing and potential dog owners when deciding between candidate breeds and/or individuals of different ages. Moreover, more complex forms of life table modelling can be used to support studies that quantify the burden of diseases on dog health and welfare^2,21^. When a disease leads to the death of a dog, this dog foregoes the potential remaining lifespan that it would have lived without that disease. Thus, the burden of the disease increases when a longer period of remaining life lost is caused by a disease, and this information about the life lost can be supplied by a dog life table.

The current study aimed to develop the first life tables for the UK companion dog population and dogs of different traits, including sex and some breeds. The study aimed to use a large data resource provided by the VetCompass™ Programme^22^ to access death information from the records of dogs under veterinary care in the UK. The resulting life tables could improve our understanding of longevity-related demographics of the dog population in the UK, whilst ultimately contributing to the improved health and welfare of dogs worldwide.

## Materials and methods

The sampling frame of the current study included all dogs under primary veterinary care at clinics participating in the VetCompass™ Programme during 2016 (i.e., dogs with at least one clinical record in 2016). The VetCompass™ Programme collates de-identified electronic patient record (EPR) data from primary-care veterinary practices in the UK for epidemiological research (VetCompass, 2019). Data fields available for VetCompass™ researchers include a unique animal identifier along with breed, date of birth, sex, neuter status and bodyweight, as well as clinical information from free-form text clinical notes, summary diagnosis terms^23^ and treatment with relevant dates. Dog breeds recognised by any of the Kennel Club (KC), the American Kennel Club and the Australian National Kennel Council were considered ‘purebred’ while all others (apart from those without any breed information) were considered ‘crossbred’^24,25^. Based on their breed, purebred dogs were classified into one of the KC breed groups (Gundog, Hound, Pastoral, Terrier, Toy, Utility and Working) or as non-KC recognised (The Kennel Club, 2019).

To identify the analytic dataset of deceased dogs for the current study, the EPRs were initially screened for candidate death cases, including dogs that were euthanased, died unassisted or whether the mechanism of death was unrecorded, using a range of search terms in the clinical note field (search terms: euth, pts*, crem*, ashes, pento*, casket, beech, decease*, death, “put to sleep”, doa, died, killed, “home bury” ~ 1, [“bury” and “home”]) and treatment field (search terms: euth*, pento*, crem*, casket, scatter, beech). The candidate cases were randomly ordered and the clinical notes of a subset of candidates were manually reviewed in detail to evaluate for case inclusion. Case inclusion criteria as a confirmed death required evidence in the EPR that the dog had died at any date from January 1st 2016 to July 31st 2020. Animals without information about sex were excluded from the final sample.

After descriptive statistics for summarising the demographics of the sample were conducted, a hypothetical cohort life table for the UK companion dog population was constructed with all dogs in the dataset^8^. Life tables for subpopulations (mentioned below) were also built: all life tables needed to have a minimum of 3 dogs in each given year interval and 11 dogs at the last year interval. A minimum of 3 dogs in each given year interval was decided to ensure a sample variance that takes the advantage of averaging (i.e., the denominator will be > 1) for “mean fraction of last year of life lived by dogs died in [x, x + 1)” (${\widehat{a}}_{x}$). Because the estimation of life expectancy at each year interval takes into account all dogs died at that age and after, the number of dogs at a single year interval does not play a major role in the estimation of 95% CI of the life expectancy. Thus, we only set the number limit of dogs for the last year interval. Based on the criteria, life tables were also constructed for (a) male and female dogs, (b) neutered and entire dogs for both sexes (c) dogs of different KC breed groups (Gundog, Hound, Pastoral, Terrier, Toy, Utility, Working, and non-KC recognised), and (d) crossbred and 18 breeds of dogs. All the life tables were complete life tables (i.e., life tables with an age interval of 1 year), except for the final interval which could extend beyond one year.

Table 1 presents the parameters in the life table and their definition and equations. The life expectancy at age 0 equates to the mean age at death of dogs across all ages.

**Table 1:** Parameters used in a life table.

Parameter	Definition	Equation
${d}_{x}$	Number of dogs dying in the year interval [x, x + 1)	
${l}_{x}$	Number of dogs living at year x ($n$ denotes the starting point for the final year interval)	$\sum_{i=x}^{n}{d}_{i}$
${\widehat{q}}_{x}$	Probability of dogs dying in the year interval [x, x + 1)	${\widehat{q}}_{x}={d}_{x}/{l}_{x}$
${\widehat{a}}_{x}$	Mean fraction of last year of life lived by dogs died in the year interval [x, x + 1)	${\widehat{a}}_{x}=\frac{\sum ({lifespan}^{*} -x)}{{d}_{x}}$
${L}_{x}$	Number of dog-years lived in the year interval [x, x + 1)	${L}_{x}={(l}_{x}-{d}_{x})+{\widehat{a}}_{x}*{d}_{x}$
${T}_{x}$	Number of dog-years lived beyond year x	$\sum_{i=x}^{n}{L}_{i}$
${\widehat{e}}_{x}$	Life expectancy at year x	${\widehat{e}}_{x}={T}_{x}/{l}_{x}$

*Lifespan of dogs dying in the year interval [x, x + 1).

Data cleaning (including removal of dogs (a) died before January 1st 2016 or after July 31st 2020, (b) with negative lifespan, (c) without birth or mortality information, (d) without sex information) and management were performed in Microsoft Excel 2013 (Microsoft Corp.) and in R programme version 4.0.2 in RStudio interface version 1.3.1073^26,27^. Descriptive analyses were facilitated by the “tidyverse” package^28^. Life table construction was performed in R, and the 95% confidence interval for life expectancy at different years was generated using empirical bootstrapping with 10,000 iterations^29^. An iteration of the life table would be taken into the estimation of 95% confidence interval only if it met the criteria for a life table stated above. R codes to generate a complete cohort life table and the confidence interval can be found online: https://github.com/kendyteng/OpenAccess/tree/main/lifetable_dog_vc_2016.

### Ethics approval and consent to participate

Ethics approval was obtained from the RVC Ethics and Welfare Committee (SR2018-1652). No human data were included in the current study.

## Results

### Demography

The sampling frame included 876,039 dogs with at least one clinical record during 2016 from 886 clinics in the VetCompass™ database. The geographic spread of the clinics with available postcode data included England (90.4%), Scotland (3.9%), Wales (3.7%), Northern Ireland (1.7%) and Channel Islands (0.3%). Initial screening identified 97,860 candidate death cases at any date from 1st January 2016 to 31st July 2020. Following a manual review of 32,390 (33.1%) of the candidate cases and excluding 72 dogs without a record of sex, the current study analysis included 30,563 (94.4% of candidates) confirmed deceased dogs. The success rate of the combined search terms to correctly identify deceased dogs was 94.6% (30,635/32,390).

Among the 30,563 dogs, 14,574 (47.7%) were female. There were 17,546 (57.4%) neutered dogs, of which 61.4% (n = 8951) were female. There were 23,963 (78.4%) purebred dogs, 6511 (21.3%) crossbred dogs, and 89 (0.3%) dogs without recorded breed information. Among 23,414 dogs of KC-recognised breeds, there were 5354 (22.9%) Gundogs, 1329 (5.7%) Hounds, 2451 (10.5%) Pastorals, 6055 (25.9%) Terriers, 3334 (14.2%) Toys, 2707 (11.6%) Utilities and 2184 (9.3%) Workings. In total, the analytic dataset included 263 ‘purebred’ breeds along with a ‘crossbred’ grouping. The number and percentage of each breed is in Supplementary File 1. There were 18 breeds included in the life table analyses, accounted for 50.6% of the population. The breeds were: American Bulldog (n = 126), Beagle (n = 171), Border Collie (n = 938), Boxer (n = 831), Cavalier King Charles Spaniel (n = 861), Chihuahua (n = 453), Cocker Spaniel (n = 1063), English Bulldog (n = 476), French Bulldog (n = 229), German Shepherd Dog (n = 1097), Husky (n = 153), Jack Russell Terrier (n = 1614), Labrador Retriever (n = 2481), Pug (n = 196), Shih-tzu (n = 635), Springer Spaniel (n = 785), Staffordshire Bull Terrier (n = 2347), and Yorkshire Terrier (1039). There were 5188 (17.0%) dogs recorded with health insurance.

### Life table

Table 2 shows the overall life table for the UK companion dog population. The life expectancy at age 0 for UK companion dogs was 11.23 (95% CI: 11.19–11.27) years, with life expectancy decreasing with age. The probability of death at each year interval increased with age with an exception of year interval 1–2 (0.017) to 2–3 (0.016). The probability of death within each year interval remained at or below 0.02 before year 5, and the increase in the probability became prominent after around year 6–7.

**Table 2:** Cohort life table of dogs under primary veterinary care in the UK.

Age (year) [x, x + 1)	Number of dogs died in [x, x + 1) (${d}_{x}$)	Number of dogs living at x (${l}_{x}$)	Probability of dogs dying in [x, x + 1) (${\widehat{q}}_{x}$)	Mean fraction of last year of life lived by dogs died in [x, x + 1) (${\widehat{a}}_{x}$)	Number of dog-years lived in [x, x + 1) (${L}_{x}$)	Number of dog-years lived beyond x (${T}_{x}$)	Life expectancy at x (${\widehat{e}}_{x}$)
0–1	514	30,563	0.017	0.42	30,263.84	343,221.39	11.23 (11.19–11.27)
1–2	481	30,049	0.016	0.52	29,819.18	312,957.55	10.41 (10.37–10.46)
2–3	482	29,568	0.016	0.45	29,304.56	283,138.37	9.58 (9.54–9.61)
3–4	489	29,086	0.017	0.48	28,829.65	253,833.80	8.73 (8.69–8.76)
4–5	559	28,597	0.020	0.48	28,304.73	225,004.16	7.87 (7.83–7.90)
5–6	677	28,038	0.024	0.47	27,679.76	196,699.43	7.02 (6.98–7.05)
6–7	892	27,361	0.033	0.46	26,883.05	169,019.67	6.18 (6.14–6.21)
7–8	1254	26,469	0.047	0.51	25,853.71	142,136.63	5.37 (5.34–5.40)
8–9	1730	25,215	0.069	0.50	24,344.36	116,282.92	4.61 (4.58–4.64)
9–10	2265	23,485	0.096	0.49	22,333.50	91,938.56	3.91 (3.89–3.94)
10–11	2852	21,220	0.134	0.48	19,746.67	69,605.06	3.28 (3.25–3.31)
11–12	3449	18,368	0.188	0.50	16,637.57	49,858.39	2.71 (2.69–2.74)
12–13	3645	14,919	0.244	0.47	13,001.49	33,220.82	2.23 (2.20–2.25)
13–14	3785	11,274	0.336	0.48	9303.98	20,219.32	1.79 (1.77–1.82)
14–15	3126	7489	0.417	0.44	5743.64	10,915.34	1.46 (1.43–1.48)
15–16	2249	4363	0.515	0.43	3070.22	5171.70	1.19 (1.15–1.22)
16–17	1253	2114	0.593	0.39	1348.90	2101.48	0.99 (0.95–1.03)
17–18	542	861	0.630	0.38	522.78	752.58	0.87 (0.82–0.93)
18–19	218	319	0.683	0.32	170.71	229.81	0.72 (0.64–0.80)
19–20	78	101	0.772	0.33	48.41	59.09	0.59 (0.47–0.71)
20 and over	23	23	1.000	0.46	10.68	10.68	0.46 (0.26–0.70)

Each column is a feature of a typical life table.

**Figure 1:**
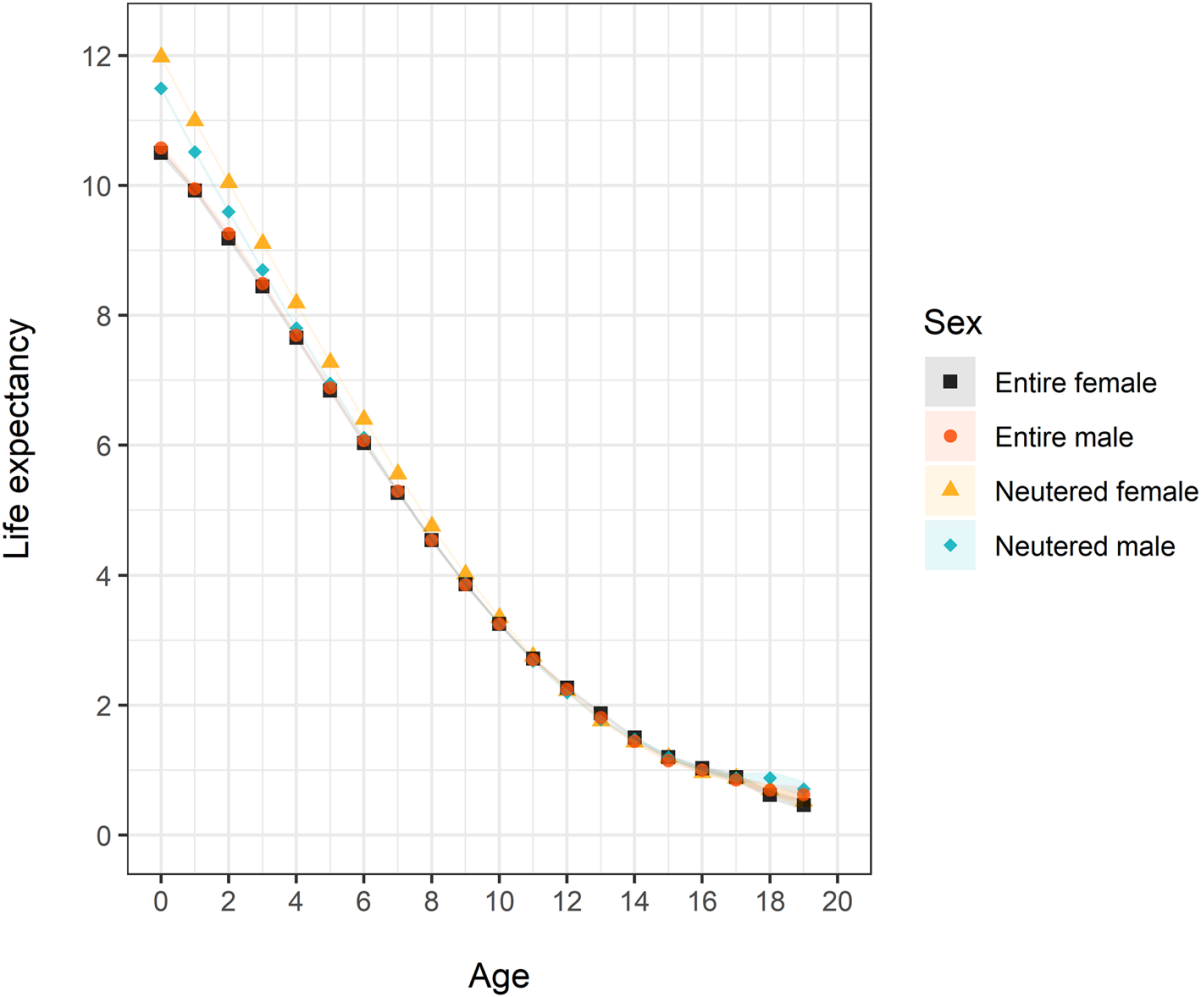
Life expectancy and the 95% confidence interval for female and male dogs at different ages (year) under primary veterinary care in the UK.

Female dogs (11.41; 95% CI: 11.35–11.47) had a longer life expectancy than male dogs (11.07; 95% CI: 11.01–11.13) at age 0 (Tables S1 and S2 in Supplementary File 2). This trend towards greater annual female life expectancy persisted until individuals were 12 years of age, after which the life expectancy of both sexes became similar. When adding neuter status into consideration, a substantially higher probability of death in entire female (year 10 and before) and male (year 4 and before) than their neutered counterparts was observed (Tables S3 to S6 in Supplementary File 2). Entire females (10.50; 95% CI: 10.38–10.61) and males (10.58; 95% CI: 10.48–10.68) had a similar life expectancy at age 0 and life expectancy trajectory (Fig. 1), whereas both neutered females (11.98; 95% CI: 11.91–12.04) and males (11.49; 95% CI: 11.42–11.57) had an elevated life expectancy at age 0 when compared to their non-neutered counterparts, especially females.

**Figure 2:**
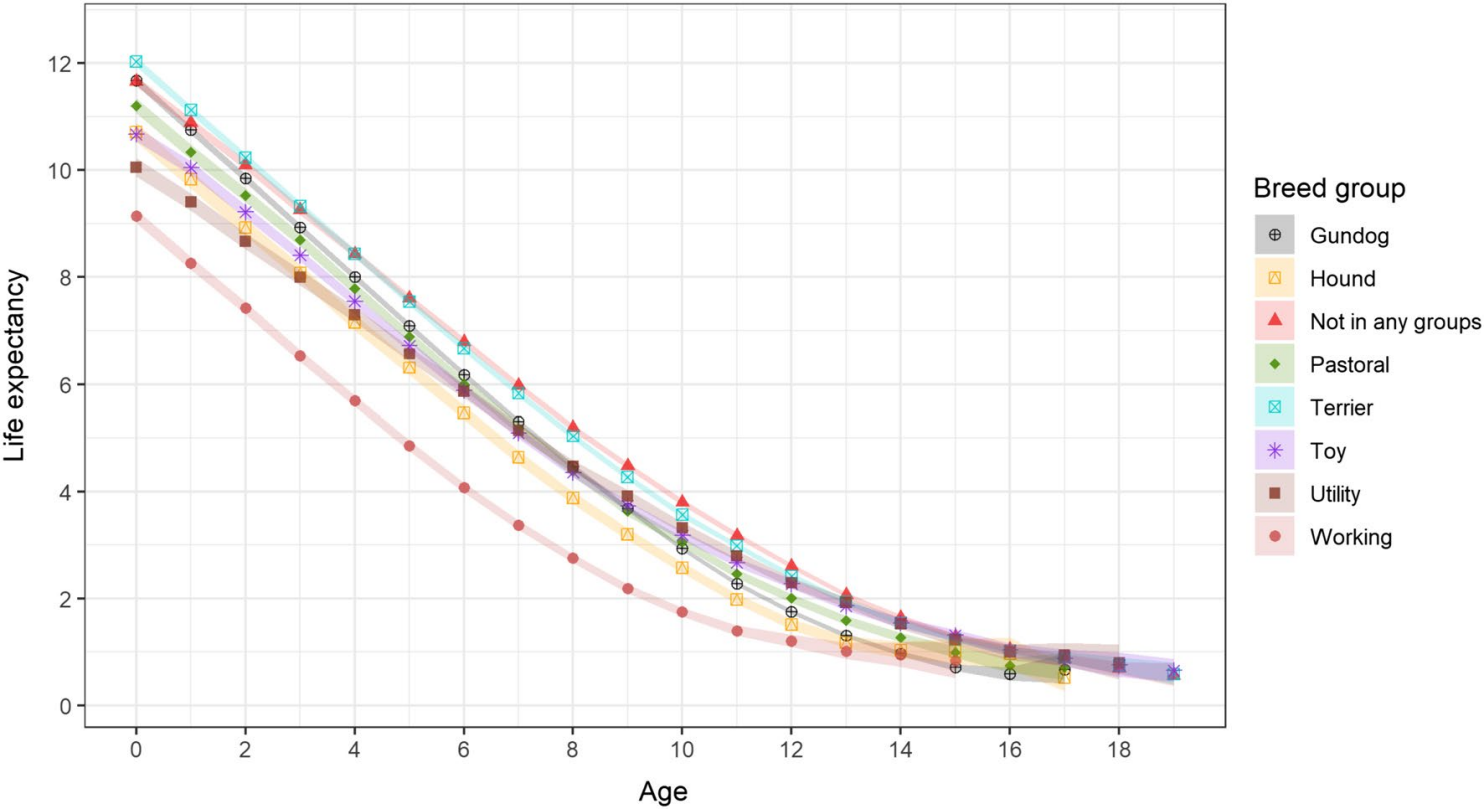
Life expectancy and the 95% confidence interval for dogs of different Kennel Club breed groups under primary veterinary care in the UK.

Among the KC breed groups and dogs of breeds not recognised by the KC, Terrier had the longest life expectancy at age 0 at 12.03 (95% CI: 11.94–12.2) years, followed by Gundog (11.67 years; 95% CI: 11.59–11.76), non-KC recognised dogs (11.66 years; 95% CI: 11.56–11.76), Pastoral (11.20 years; 95% CI: 11.06–11.35), Hound (10.71 years; 95% CI: 10.53–10.89), Toy (10.67 years; 95% CI: 10.54–10.81), and Utility (10.06 years; 95% CI: 9.89–10.23) (Fig. 2). Working dogs’ life expectancy was shorter than all the other groups at all ages, with a life expectancy of 9.14 (95% CI: 9.01–9.27) years at age 0. However, comparative patterns of life expectancy between the breed groups at age 0 were not necessarily maintained later into life. For instance, Hound and Toy groups had a similar life expectancy at age 0 but diverged soon after this to reach a difference of 0.76 years (higher in Toy) at year 12. The life table of Hound ended at year 17 with a life expectancy of 0.52 (95%CI: 0.27–0.77) years, whereas Toy dogs at year 19 still can be expected to live for 0.66 (95%CI: 0.44–0.87) years. Supplementary File 2 (Tables S7 to S14) contains the life tables for the breed groups.

**Figure 3:**
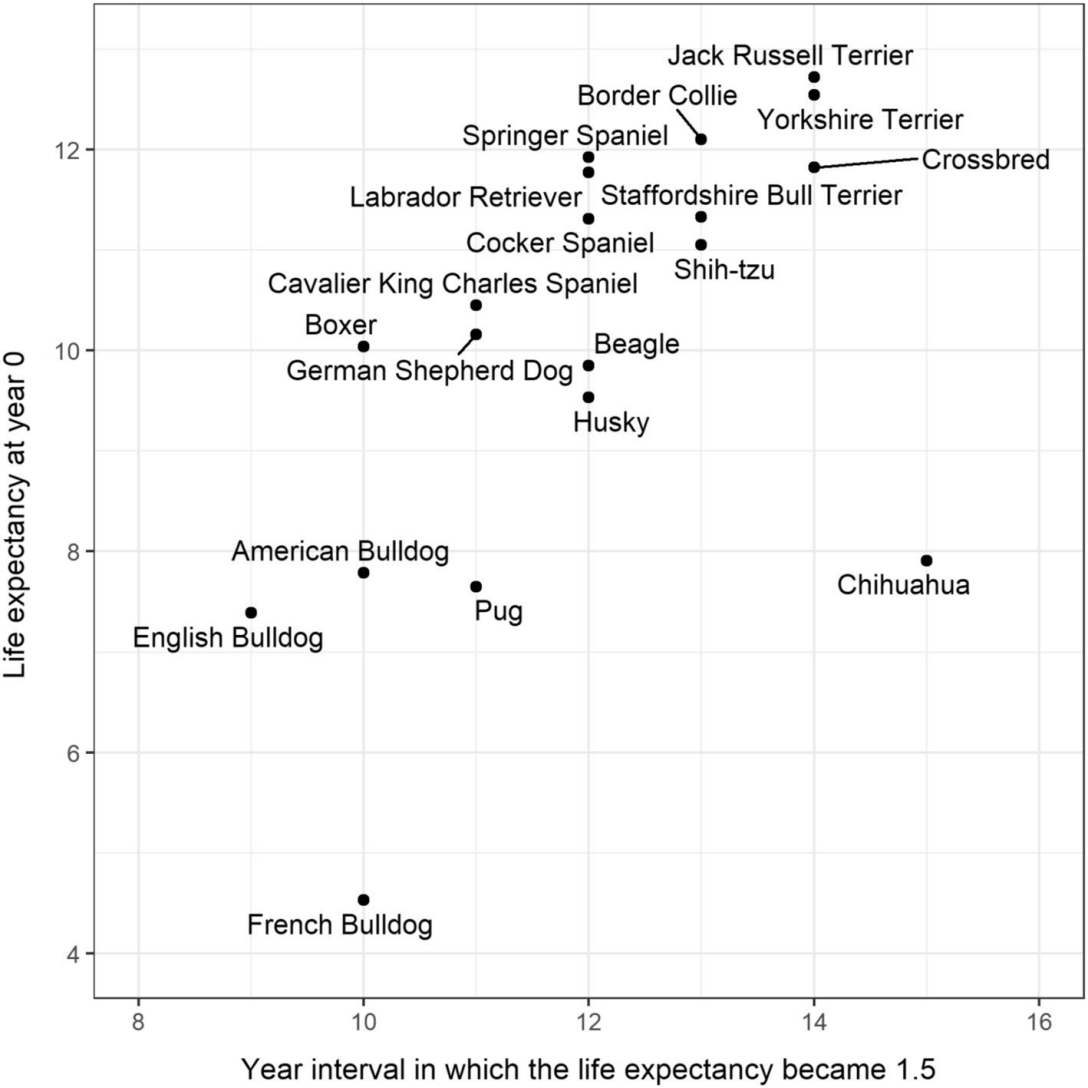
Relation between the life expectancy at year 0 and year interval in which the life expectancy became 1.5 in 18 breeds and crossbred under primary veterinary care in the UK.

Life tables for the 18 breeds and crossbred varied widely (Table 3) and can be found in Supplementary File 2 (Tables S15 to S33). The last age of the life tables ranged from 11 in French Bulldogs to 19 in Jack Russell Terrier. Jack Russell Terrier had the greatest life expectancy at age 0 at 12.72 (95% CI: 12.53–12.90) years, followed by Yorkshire Terrier (12.54 years; 95% CI: 12.30–12.77), Border Collie (12.10 years; 95% CI: 11.85–12.33) and Springer Spaniel (11.92 years; 95% CI: 11.69–12.13). Compared to other breeds, many brachycephalic breeds (i.e., breeds of dogs with a short, flat face) had a relatively short life expectancy at age 0, with French Bulldog having the shortest at 4.53 (95% CI: 4.14–5.01) years, 2.86 years less than the value for English Bulldog (7.39 years; 95% CI: 7.08–7.69). To explore the longevity of the dogs of different breeds, we examined the earliest age at which the life expectancy dropped below 1.5 years (1.5 years was chosen because the life expectancy at the last year of all breeds was less than this value; Fig. 3). The life expectancy dropping below 1.5 years occurred in Chihuahuas at year 15–16, followed by Jack Russell Terrier, crossbred dogs and Yorkshire Terrier at year 14–15. English Bulldog was the earliest to reach the life expectancy of 1.5 years (year 9–10), followed by Boxer, French Bulldog and American Bulldog at year 10–11.

**Table 3:** Key statistics extracted from the life tables of 18 individual dog breeds and of crossbred, including the life expectancy at age 0 and at the last age, using the data of dogs under primary veterinary care in the UK.

Breed	Life expectancy ($\widehat{e}$) at age 0 and the 95% confidence interval (CI)	Last age (year) in the life table	$\widehat{e}$ at the last age (year) in the life table and the 95% CI	Year interval when $\widehat{e}$ become 1.5	Number of dogs in the life table
Jack Russell Terrier	12.72 (12.53–12.90)	19	0.66 (0.39–0.97)	14–15	1620
Yorkshire Terrier	12.54 (12.30–12.77)	18	0.74 (0.45–1.05)	14–15	1042
Border Collie	12.10 (11.85–12.33)	17	0.68 (0.42–1.00)	13–14	942
Springer Spaniel	11.92 (11.69–12.13)	16	0.73 (0.47–1.03)	12–13	790
Crossbred	11.82 (11.72–11.92)	19	0.54 (0.36–0.76)	14–15	6511
Labrador Retriever	11.77 (11.67–11.89)	16	0.51 (0.34–0.71)	12–13	2500
Staffordshire Bull Terrier	11.33 (11.19–11.48)	18	0.61 (0.29–0.97)	13–14	2364
Cocker Spaniel	11.31 (11.13–11.53)	16	0.51 (0.33–0.70)	12–13	1073
Shih-tzu	11.05 (10.73–11.40)	17	0.83 (0.48–1.26)	13–14	638
Cavalier King Charles Spaniel	10.45 (10.26–10.62)	15	0.77 (0.48–1.10)	11–12	867
German Shepherd Dog	10.16 (10.00–10.370	15	0.79 (0.50–1.14)	11–12	1110
Boxer	10.04 (9.85–10.21)	15	0.83 (0.29–1.46)	10–11	836
Beagle	9.85 (9.17–10.24)	14	1.08 (0.62–1.59)	12–13	172
Husky	9.53 (8.71–9.88)	14	1.02 (0.49–1.60)	12–13	154
Chihuahua	7.91 (7.48–8.39)	16	1.07 (0.67–1.52)	15–16	458
American Bulldog	7.79 (7.16–8.33)	12	1.48 (0.95–2.03)	10–11	129
Pug	7.65 (6.99–8.20)	13	1.17 (0.75–1.63)	11–12	197
English Bulldog	7.39 (7.08–7.69)	13	0.59 (0.35–0.82)	9–10	478
French Bulldog	4.53 (4.14–5.01)	11	1.39 (0.84–1.98)	10–11	232

The probability of death was lower in year 0–1 than year 1–2 in most breeds [American Bulldog (0.024; 0.065), Border Collie (0.012; 0.020), Boxer (0.005; 0.016), English Bulldog (0.040; 0.055), Cocker Spaniel (0.012; 0.013), French Bulldog (0.131; 0.136); German Shepherd Dog (0.013; 0.020), Husky (0.026; 0.047), Jack Russell Terrier (0.009; 0.009); Labrador Retriever (0.007; 0.009), Springer Spaniel (0.006; 0.006) and Staffordshire Bull Terrier (0.011; 0.012)]. Some breeds, including American Bulldog, Chihuahua, English Bulldog, French Bulldog, Husky and Pug had a probability of death before reaching adulthood (before year 2^30^) much higher than the overall dogs (0.017 in year 0–1 and 0.016 in year 1–2).

## Discussion

This study presents the first cohort life tables for the UK dog population and dogs of different characteristics, including sex, neuter status, KC grouping and 18 breeds and crossbred dogs. These tables offer information about annual life expectancy and annual probability of death, which has been unavailable from conventional longevity studies in UK companion dogs to date. With the ongoing accumulation and accessibility of death information from Big Data resources such as VetCompass in the future, the construction of life tables for increasing numbers of breeds of dogs and also for other companion animal species should expand. The current study provides proof of concept for the development of (hypothetical) cohort life table construction in companion animals and also shares open-access R codes to contribute to these wider purposes (see “Materials and methods”).

Based on the mathematical and biological plausibility, a valid life table should exhibit the highest life expectancy at age 0 which decreases with age^8,15,31^. The probability of death may be higher in infancy as the immune system continues to mature in the postnatal period, for both humans and dogs^32^. In dogs, the immune system takes approximately one year for full maturity^32^. In the most recent life tables of humans in the UK, Australia and the US, the probability of death appeared the lowest in ages of 7–11 years (i.e., the onset of senescence) before showing an increasing trend until the end of the table (i.e., life)^15,33,34^. Our overall life table in dogs satisfied the condition of decreasing life expectancy and had the lowest annual probability of death (${\widehat{q}}_{x})$ before reaching adulthood at year 2^30^, similar to the life tables in humans. Some life tables constructed in the current study did not follow this trend in the probability of death, for example, the life tables for neutered males and females. These life tables will be discussed below.

The life expectancy at age 0 reported in the current study for dogs under primary veterinary care in the UK in 2016 was 11.23 years (11.19–11.27), 2.47 years shorter than the life expectancy at age 0 (both 13.7 years) in the two life tables of Japanese dogs constructed using pet insurance and pet cemetery data, discussed above^10,17^. Differing data sources for the study populations might partially contribute to this substantial variation. Insured dogs may represent a subset of dogs that live longer than those without insurance, as they may receive more or additional veterinary care, due to alleviated economic constraints on the owners^35,36^. Moreover, dog breeds of high disease risk such as brachycephalic breeds might be under-represented in some insurance data than the general population due to the elevated cost to insure and special rules of reimbursement applied to these breeds in some insurance companies^37,38^. Breed demographics are likely to differ between these two counties. Breeds of toy or small size have a longer life expectancy than larger-sized dogs and are more common in Japan than in the UK^1,5,10,17^. In contrast, breeds of large and medium sizes, as well as brachycephalic dogs, are more popular in the UK^39^, which present shorter life expectancies. These demographic differences will influence country-level life expectancy estimates at age 0. However, it appeared that even within the same breeds, the life expectancy of dogs in Japan was considerably higher than those in the UK^10^, such as Labrador Retriever (UK = 11.77 and Japan = 14.1 years), Shih Tzu (UK = 11.05 and Japan = 15.0 years), Beagle (UK = 9.85 and Japan = 14.8 years), Pug (UK = 7.65 and Japan = 12.8 years), and French Bulldog (UK = 4.53 and Japan = 10.2 years). Variation in estimates may be partly due to sampling effort, as the life expectancy tables from Japan were created using a smaller sample size.

While female dogs (11.41; 95% CI 11.35–11.47) showed a longer life expectancy at age 0 than male dogs (11.07; 95% CI 11.01–11.13), this phenomenon was moderated by neuter status. Entire animals of both sexes showed similar trajectories of life expectancy from age 0 onwards. However, neutering was associated with an elevated life expectancy at age 0 for both sexes compared to their entire counterparts, and this longevity advantage from neutering was higher in female dogs than in male dogs. A similar survival advantage for neutered animals has been reported in several studies^1,40,41^, but most data, including the current study, generated these results by dichotomising dogs into neutered or entire without taking into account the duration of gonadal hormone exposure before the neutering. Neutered animals in these cited studies would have already lived to the age of neutering, biasing their life expectancy towards greater length, highlighted by the lowered probability of death at year 0–1 in neutered dogs. As veterinarians may often recommend early neutering for female dogs, sometimes before the start of the oestrus cycle^42^ or soon after the first cycle^43^, neutering of females may occur earlier in life than neutering for males^43^. Therefore, the gap of true life expectancy between the sexes due to neutering might be even wider than reported here. Neutering may also act as a proxy for stronger owner responsibility and better care, as it is often considered responsible dog ownership. Thus, neutered animals may benefit from additional survival advantages related to enhanced owner care^43^. Neutering may directly affect the risks of various health conditions and therefore shift life expectancy as a result^41^. In female dogs, neutering reduces or eliminates the risk of pyometra, a potentially life-threatening condition that occurs in 2% of entire female dogs under 10 years^44^. Neutering is linked to a reduced risk of tumours within reproductive organs and various cardiovascular diseases, but an increased risk of joint disorders and several types of tumours such as lymphoma and hemangiosarcoma, especially in females^45^. Due to the complexity stated above, our life tables for neutered dogs should be interpreted with great caution.

For the 18 breeds and the crossbred category, the number of years contained in the tables, life expectancy and the probability of death at different ages varied widely. Relatively shorter life expectancy at specific year points can be taken as evidence that events and processes eventually leading to mortality are occurring earlier in life in these populations than some other populations, so these populations may have generally poorer health than some other populations^30,46^. If the distribution of external factors that may lead to differences in the life expectancy and the probability of death (e.g. severe disease epidemics and/or substantial differences in the level of veterinary and owner care) do not depend on the breeds, it may be safe to assume that part of the life expectancy difference is contributed by internal factors driven by the genetic make-up of the breeds. Breed predisposition to particular disorders is a well-identified phenomenon^47^. Breeds that show high levels of potentially life-threatening predispositions that start early in life are likely to have a higher probability of death at younger ages and therefore a decreased life expectancy. Indeed, four brachycephalic breeds (French Bulldog, English Bulldog, Pug and American Bulldog) that showed the shortest life expectancy at year 0 of all 18 breeds in our results are also reported with several predispositions to life-limiting disorders that occur early in life, such as brachycephalic obstructive airway syndrome, spinal disease and dystocia^48–51^.

Lifespan variations between breeds were explored by examining the association between the breed life expectancy at age 0 and the year interval at which the life expectancy reached 1.5. Generally, life expectancy at age 0 and the year interval in which the life expectancy became 1.5 were positively associated, as would be expected: breeds that lived longer were also older when they reached an age with 1.5 years of life expectancy. However, although Chihuahuas showed a life expectancy at age 0 of only 7.91, the year interval in which the life expectancy became 1.5 was year 15–16, the highest of all the breeds, indicating a high variation of lifespans among Chihuahuas. A lowered life expectancy at age 0 suggests an increase in mortality of younger-aged dogs (whose mortality is usually low), and the life expectancy becoming 1.5 years at a later age implies more dogs also living to advanced age; both increase the variation of lifespans^11^. In our results, the probability of death before year 13–14 was higher (and much higher before year 4) in Chihuahua than for dogs overall, which became lower after that. It was also observed in French Bulldogs that a low life expectancy at age 0 (4.55 years) and relatively old year interval when the life expectancy became 1.5 years (year 10–11, more than twice as long as the life expectancy at age 0) and that their probability of death was rather uniform across all ages. However, although the high variation of lifespans of French Bulldogs could be due to high health risks in early life^49–52^ and a relatively small sample size (n = 232), it may partly be attributed to recent soaring popularity^53^. The number of KC registered French Bulldogs in the UK rose steeply from 2771 in 2011 to 39,266 in 2020^39^, suggesting that the population of French Bulldogs (and other breeds sharing a similar rising trend in popularity) in our dataset are biased towards younger dogs that contribute proportionately more deaths in younger ages in the life table. In contrast, breeds with a decreasing trend in popularity may have an underestimated probability of death at younger ages, resulting in overestimated life expectancy. Previous studies have also shown the rising popularity of certain breeds and the association of rising popularity with lower median age^54–60^. Hypothetical cohort life tables are more susceptible to the influence of population instability, which is common in dogs due to sudden and dramatic fad-like changes in breed popularity^61^. This can be avoided by implementing current or real cohort life tables instead if such data were available.

Thirteen of the 18 breeds had a lower probability of death in year 0–1 than year 1–2 in the life tables, some slightly and others substantially. This finding goes against the evidence that mortality is higher in puppy (0–26 weeks) and juvenile (27–52 weeks) periods than young adult period (1–2 years) and empirical results in human life tables^15,33,34^. This may, in part, be due to substantial puppy mortality occurring before individuals can be registered to a primary veterinary clinic resulting in these deaths not appearing in the current dataset. A more accurate estimation of life expectancy from birth would be possible if all the currently unavailable puppy mortality information could be recovered.

Life tables in companion animals offer extensive applications. Similar to a common application for human life table studies, comparison between life tables can support deeper insight into the health of dogs of differing demographics such as sex and breed over time and space^62,63^. When life tables are generated periodically for a specific population, changes in the life expectancy and the probability of death at specific ages can indicate changes in the general health and welfare of the population. Comparison of life tables among the populations of different traits such as breed or conformation can also identify less healthy or more vulnerable populations^62^, as demonstrated in our study (especially for breeds). Advanced life table modelling can offer useful information allowing the quantification of disease burden on health and welfare in human and companion animals^2,64,65^. Quantification of disease burden is important because it can assist with the prioritisation of health conditions for targeted reform^66–68^. Coupling these findings with cost-effective analysis on disease prevention and control can assist to allocate resources to priority health conditions and achieve efficient improvements in the health and welfare of the overall population^69^. The value of disease burden quantification has been demonstrated by the Global Burden of Disease project of the World Health Organization to improve human health^70^. The Global Burden of Disease uses the Disability-Adjusted Life Year framework that incorporates life tables as part of the methodology to quantify the burden of many diseases (369 diseases and injuries in 2019)^64,65^. The Disability-Adjusted Life Year has been adapted into the Welfare-Adjusted Life Years (WALY) to quantify the burden of common diseases on dogs' welfare^2^. The WALY constitutes two elements, (a) years lived with impaired welfare, which is the years having a certain disease weighted by its severity and (b) years of life lost due to the premature death caused by the disease or resulting assisted death. Future life table modelling that accounts for comorbidity and demographics of dogs can offer information about years of life lost based upon the life expectancy at the age of death for the individual animal affected.

Life tables highlight the value of interpreting life expectancy annually, especially at older ages, where differences in life expectancy between ages become narrower. Thus, the current authors propose that life table literacy is important for veterinary professionals, shelter staff, and dog owners because it can optimise decision-making and subsequently can positively impact dog welfare. Life table literacy will promote realistic expectations for the life expectancy of dogs at different ages, helping to make treatment plans for illness and end of life decisions. Shelters and charities can also incorporate this information in the adoption process ensuring that potential dog owners understand the expected length of ownership commitment required for dogs of different breeds, ages, and neuter status.

With the foundations for canine life table science built by the current work, we hope to generate further examples of life tables for both dogs and cats using the VetCompass data in the future. The current study provides a proof of concept that can support future research looking to construct life tables for dogs and cats as a periodic recurring endeavour. Consequently, changes in the life expectancy, mortality, and health of companion dogs and cats can be tracked similarly to how it is in human demography^15,16^. For future life table construction, we hope to incorporate other sources of information such as KC annual registry data and dog insurance data with further modelling, which will help to produce even more accurate life tables^71^.

This study had some additional limitations to those discussed above. Firstly, the high frequency of euthanasia in companion dogs highlights the potentially underestimated life expectancy compared to unassisted death^72^. This is especially biased by euthanasia undertaken for non-life-threatening reasons such as undesirable behaviours, economic reasons or convenience^35,73^. Consequently, differing cultures between countries for euthanasia in dogs might substantially influence national life tables. Another limitation is the sole inclusion of primary veterinary practice-attending dogs. Thus, our results might be less representative of unowned dogs or dogs not attending veterinary clinics. Also, some dogs that died at home or in emergency out-of-hours clinics might be excluded from the current data, although the data did capture all deaths away from the clinics that were reported by owners to the veterinary clinics at any time. Lastly, the sample sizes for some of the 18 breeds (e.g. American Bulldogs, Beagle, English Bulldog, French Bulldog, Husky and Pug) were relatively small, resulting in life tables with reserved confidence.

## Conclusion

The current study has produced the first life tables for dogs in the UK, reporting annual life expectancy and probability of death for the UK companion dog population, dogs of different sex and neuter status, breed groups and also for 18 breeds and crossbred dogs. We report an elevated life expectancy in neutered dogs compared to entire dogs and wide variation in life expectancy between breeds, with Jack Russell Terrier and Yorkshire Terrier having the highest and some brachycephalic breeds showing the lowest life expectancy at age 0. The construction and application of life tables offers great potential for companion animal health and welfare sciences but is still in its infancy. Life tables generated in the current study promote not only a better understanding of the life trajectory of dogs but also offer several applications for the veterinary profession and research to improve the health and welfare of dogs.

## Supplementary Information

Supplementary Information 1.

Supplementary Tables.

## Data Availability

The datasets generated and analysed during the current study are publicly available on the RVC Data Repository at https://rvc-repository.worktribe.com/output/1558210. R codes for cohort life table construction and all life tables generated by the current study can be found at https://github.com/kendyteng/cohort_lifetable.

## Abbreviations

UK: United Kingdom
CI: Confidence interval
EPR: Electronic patient record
IQR: Interquartile range
KC: Kennel Club
WALY: Welfare-Adjusted Life Years

## References

[1] O’Neill DG, Church DB, McGreevy PD, Thomson PC, Brodbelt DC (2013). Longevity and mortality of owned dogs in England. Vet. J..

[2] Teng KT (2018). Welfare-Adjusted Life Years (WALY): A novel metric of animal welfare that combines the impacts of impaired welfare and abbreviated lifespan. PLoS ONE.

[3] Fleming JM, Creevy KE, Promislow DEL (2011). Mortality in North American dogs from 1984 to 2004: An investigation into age-, size-, and breed-related causes of death. J. Vet. Intern. Med..

[4] Bonnett BN, Egenvall A (2010). Age patterns of disease and death in insured Swedish dogs, cats and horses. J. Comp. Pathol..

[5] Adams VJ, Evans KM, Sampson J, Wood JLN (2010). Methods and mortality results of a health survey of purebred dogs in the UK. J. Small Anim. Pract..

[6] Lewis TW, Wiles BM, Llewellyn-Zaidi AM, Evans KM, O’Neill DG (2018). Longevity and mortality in Kennel Club registered dog breeds in the UK in 2014. Canine Genet. Epidemiol..

[7] Chiang CL (1972). On constructing current life tables. JASA.

[8] Chiang CL (1984). The Life Table and Its Applications.

[9] Feuer EJ (1993). The lifetime risk of developing breast cancer. J. Natl. Cancer Inst..

[10] Inoue M, Kwan NCL, Sugiura K (2018). Estimating the life expectancy of companion dogs in Japan using pet cemetery data. J. Vet. Med. Sci..

[11] Vaupel JW, Zhang Z, van Raalte AA (2011). Life expectancy and disparity: An international comparison of life table data. BMJ Open.

[12] Centers for Disease Control and Prevention. Products-Life Tables-Homepage. https://www.cdc.gov/nchs/products/life_tables.htm (2019).

[13] Lledó J, Pavía JM, Morillas FG (2017). Assessing implicit hypotheses in life table construction. Scand. Actuar. J..

[14] Soneji S, King G (2012). Statistical security for social security. Demography.

[15] Office for National Statistics. National life tables: UK - Office for National Statistics. https://www.ons.gov.uk/peoplepopulationandcommunity/birthsdeathsandmarriages/lifeexpectancies/datasets/nationallifetablesunitedkingdomreferencetables/current (2020).

[16] Arias, E., Xu, J. & Division of Vital Statistics. United States life tables, 2017. *National Vital Statistics Reports* 66 (2019). 32501200

[17] Inoue M, Hasegawa A, Hosoi Y, Sugiura K (2015). A current life table and causes of death for insured dogs in Japan. Prev. Vet. Med..

[18] Jónás D, Sándor S, Tátrai K, Egyed B, Kubinyi E (2020). A preliminary study to investigate the genetic background of longevity based on whole-genome sequence data of two methuselah dogs. Front. Genet..

[19] Kwan NCL, Ogawa H, Yamada A, Sugiura K (2016). Quantitative risk assessment of the introduction of rabies into Japan through the illegal landing of dogs from Russian fishing boats in the ports of Hokkaido, Japan. Prev. Vet. Med..

[20] Proschowsky HF, Rugbjerg H, Ersbøll AK (2003). Mortality of purebred and mixed-breed dogs in Denmark. Prev. Vet. Med..

[21] Wang H (2020). Global age-sex-specific fertility, mortality, healthy life expectancy (HALE), and population estimates in 204 countries and territories, 1950–2019: A comprehensive demographic analysis for the Global Burden of Disease Study 2019. Lancet.

[22] VetCompass. VetCompass^TM^ Programme. http://www.rvc.ac.uk/VetCOMPASS/ (RVC Electronic Media Unit, 2019).

[23] The Venom Coding Group. VeNom Veterinary Nomenclature. http://venomcoding.org (2019).

[24] The Kennel Club. Breed Information Centre. http://www.thekennelclub.org.uk/services/public/breed/ (2019).

[25] American Kennel Club. Dog Breeds. *American Kennel Club* https://www.akc.org/dog-breeds/ (2021).

[26] R Core Team (2019). R: A Language and Environment for Statistical Computing.

[27] RStudio Team (2019). RStudio: Integrated Development for R.

[28] Wickham, H. *tidyverse: Easily install and load the ‘tidyverse’*. (2017).

[29] Efron B, Tibshirani RJ (1994). An Introduction to the Bootstrap.

[30] Harvey ND (2021). How old Is my dog? identification of rational age groupings in pet dogs based upon normative age-linked processes. Front. Vet. Sci..

[31] Szilard L (1959). On the nature of aging process. Proc. Natl. Acad. Sci. USA.

[32] Felsburg PJ (2002). Overview of immune system development in the dog: Comparison with humans. Hum. Exp. Toxicol..

[33] Arias E, Xu J (2020). United States life tables, 2018. Natl. Vital Stat. Rep..

[34] Australian Bureau of Statistics. Life tables. https://www.abs.gov.au/statistics/people/population/life-tables (2020).

[35] Boller M (2020). The effect of pet insurance on presurgical euthanasia of dogs with gastric dilatation-volvulus: A novel approach to quantifying economic euthanasia in veterinary emergency medicine. Front. Vet. Sci..

[36] Egenvall A, Nødtvedt A, Penell J, Gunnarsson L, Bonnett BN (2009). Insurance data for research in companion animals: Benefits and limitations. Acta Vet. Scand..

[37] Chaters, G., Trees, A. J. & Laing, G*. Higher Insurance Premiums Revealed for Popular Brachycephalic Breeds* https://vprf.wordpress.com/2020/07/09/higher-insurance-premiums-revealed-for-popular-brachycephalic-breeds/ (Veterinary Policy Research Foundation, 2020).

[38] Bergström A, Nødtvedt AN, Lagerstedt AS, Egenvall AA (2006). Incidence and breed predilection for dystocia and risk factors for cesarean section in a Swedish population of insured dogs. Vet. Surg..

[39] The Kennel Club. Breed registration statistics. https://www.thekennelclub.org.uk/media-centre/breed-registration-statistics/ (2021).

[40] Hoffman JM, O’Neill DG, Creevy KE, Austad SN (2018). Do female dogs age differently than male dogs?. J. Gerontol. Ser. A.

[41] Urfer SR, Kaeberlein M (2019). Desexing dogs: A review of the current literature. Animals.

[42] Hart LA, Hart BL (2021). An ancient practice but a new paradigm: Personal choice for the age to spay or neuter a dog. Front. Vet. Sci..

[43] Diesel G, Brodbelt D, Laurence C (2010). Survey of veterinary practice policies and opinions on neutering dogs. Vet. Rec..

[44] Egenvall A (2001). Breed risk of pyometra in insured dogs in Sweden. J. Vet. Intern. Med..

[45] Belanger JM, Bellumori TP, Bannasch DL, Famula TR, Oberbauer AM (2017). Correlation of neuter status and expression of heritable disorders. Canine Genet. Epidemiol..

[46] Banzato T (2019). A Frailty Index based on clinical data to quantify mortality risk in dogs. Sci. Rep..

[47] Yordy J (2020). Body size, inbreeding, and lifespan in domestic dogs. Conserv. Genet..

[48] Gough A, Thomas A, O’Neill D (2018). Breed Predispositions to Disease in Dogs and Cats.

[49] O’Neill DG (2015). Epidemiological associations between brachycephaly and upper respiratory tract disorders in dogs attending veterinary practices in England. Canine Genet. Epidemiol..

[50] O’Neill DG (2019). Canine dystocia in 50 UK first-opinion emergency care veterinary practices: Clinical management and outcomes. Vet. Rec..

[51] Liu N-C (2017). Outcomes and prognostic factors of surgical treatments for brachycephalic obstructive airway syndrome in 3 breeds. Vet. Surg..

[52] Ryan R, Gutierrez-Quintana R, ter Haar G, De Decker S (2017). Prevalence of thoracic vertebral malformations in French bulldogs, Pugs and English bulldogs with and without associated neurological deficits. Vet. J..

[53] Teng KT, McGreevy PD, Toribio J-ALML, Dhand NK (2016). Trends in popularity of some morphological traits of purebred dogs in Australia. Canine Genet. Epidemiol..

[54] O’Neill DG, Baral L, Church DB, Brodbelt DC, Packer RMA (2018). Demography and disorders of the French Bulldog population under primary veterinary care in the UK in 2013. Canine Genet. Epidemiol..

[55] O’Neill DG (2019). Disorders of Bulldogs under primary veterinary care in the UK in 2013. PLoS ONE.

[56] O’Neill DG, Darwent EC, Church DB, Brodbelt DC (2016). Demography and health of Pugs under primary veterinary care in England. Canine Genet. Epidemiol..

[57] O’Neill DG, Coulson NR, Church DB, Brodbelt DC (2017). Demography and disorders of German Shepherd Dogs under primary veterinary care in the UK. Canine Genet. Epidemiol..

[58] O’Neill DG, Seah WY, Church DB, Brodbelt DC (2017). Rottweilers under primary veterinary care in the UK: Demography, mortality and disorders. Canine Genet. Epidemiol..

[59] O’Neill DG, Butcher C, Church DB, Brodbelt DC, Gough AG (2019). Miniature Schnauzers under primary veterinary care in the UK in 2013: Demography, mortality and disorders. Canine Genet. Epidemiol..

[60] McGreevy PD (2018). Labrador retrievers under primary veterinary care in the UK: Demography, mortality and disorders. Canine Genet. Epidemiol..

[61] Herzog H (2006). Forty-two thousand and one Dalmatians: Fads, social contagion, and dog breed popularity. Soc. Anim..

[62] Andrasfay, T. & Goldman, N. Reductions in 2020 US life expectancy due to COVID-19 and the disproportionate impact on the Black and Latino populations. *PNAS* **118**, (2021). 10.1073/pnas.2014746118 PMC7865122 33446511

[63] Johnson S (2021). Extra Life: A Short History of Living Longer.

[64] Murray CJ (1994). Quantifying the burden of disease: The technical basis for disability-adjusted life years. Bull. World Health Organ..

[65] Vos T (2020). Global burden of 369 diseases and injuries in 204 countries and territories, 1990–2019: A systematic analysis for the Global Burden of Disease Study 2019. Lancet.

[66] Rioja-Lang F, Bacon H, Connor M, Dwyer CM (2019). Determining priority welfare issues for cats in the United Kingdom using expert consensus. Vet. Rec. Open.

[67] Rioja-Lang F, Bacon H, Connor M, Dwyer CM (2019). Rabbit welfare: Determining priority welfare issues for pet rabbits using a modified Delphi method. Vet. Rec. Open.

[68] Summers JF (2019). Health-related welfare prioritisation of canine disorders using electronic health records in primary care practice in the UK. BMC Vet. Res..

[69] Neumann PJ (2016). A systematic review of cost-effectiveness studies reporting cost-per-DALY averted. PLoS ONE.

[70] Institute for Health Metrics and Evaluation. Global Burden of Disease (GBD 2019). http://www.healthdata.org/gbd/2019 (Institute for Health Metrics and Evaluation, 2021).

[71] Dicker D (2018). Global, regional, and national age-sex-specific mortality and life expectancy, 1950–2017: A systematic analysis for the Global Burden of Disease Study 2017. Lancet.

[72] Pegram C (2021). Proportion and risk factors for death by euthanasia in dogs in the UK. Sci. Rep..

[73] Boyd C (2018). Mortality resulting from undesirable behaviours in dogs aged under three years attending primary-care veterinary practices in England. Anim. Welf..

