# Supplementary material for: Life tables of annual life expectancy and mortality for companion dogs in the United Kingdom
Source: Sci Rep. 2022 Apr 28;12:6415. doi: 10.1038/s41598-022-10341-6 (PMC9050668; doi:10.1038/s41598-022-10341-6)
Supplement: Supplementary file 2 — Supplementary Tables. [file 41598_2022_10341_MOESM2_ESM.pdf]

Supplementary File 2. Life tables for dogs of different traits under primary veterinary care in the United Kingdom (UK).

Table S1. Cohort life table of female dogs under primary veterinary care in the UK.

Age (year) [x, x+1)	Number of dogs died in [x, x+1) (d_x)	Number of dogs living at x (l_x)	Probability of dogs dying in [x, x+1) ($\hat{q}_x$)	Mean fraction of last year of life lived by dogs died in [x, x+1) ($\hat{a}_x$)	Number of dog−years lived in [x, x+1) (L_x)	Number of dogs living at year x (T_x)	Life expectancy at year x ($\hat{e}_x$)
0−1	237	14574	0.016	0.41	14434.12	166233.05	11.41 (11.35−11.47)
1−2	188	14337	0.013	0.52	14247.29	151798.93	10.59 (10.53−10.65)
2−3	210	14149	0.015	0.45	14033.62	137551.64	9.72 (9.67−9.78)
3−4	213	13939	0.015	0.45	13821.00	123518.01	8.86 (8.81−8.91)
4−5	225	13726	0.016	0.50	13612.75	109697.01	7.99 (7.94−8.04)
5−6	298	13501	0.022	0.46	13340.71	96084.26	7.12 (7.07−7.17)
6−7	412	13203	0.031	0.48	12989.35	82743.55	6.27 (6.22−6.31)
7−8	563	12791	0.044	0.51	12515.05	69754.19	5.45 (5.41−5.50)
8−9	780	12228	0.064	0.50	11836.48	57239.15	4.68 (4.64−4.72)
9−10	1051	11448	0.092	0.50	10920.43	45402.67	3.97 (3.93−4.01)
10−11	1351	10397	0.130	0.49	9706.19	34482.24	3.32 (3.28−3.35)
11−12	1649	9046	0.182	0.50	8223.04	24776.05	2.74 (2.70−2.78)
12−13	1781	7397	0.241	0.48	6465.18	16553.01	2.24 (2.20−2.27)
13−14	1876	5616	0.334	0.48	4647.37	10087.83	1.80 (1.76−1.83)
14−15	1578	3740	0.422	0.44	2857.93	5440.46	1.45 (1.42−1.49)
15−16	1103	2162	0.510	0.43	1538.45	2582.52	1.19 (1.15−1.24)
16−17	635	1059	0.600	0.39	669.54	1044.07	0.99 (0.93−1.04)
17−18	255	424	0.601	0.37	262.18	374.53	0.88 (0.81−0.96)
18−19	121	169	0.716	0.34	88.71	112.35	0.66 (0.57−0.76)
19+	48	48	1	0.49	23.64	23.64	0.49 (0.35−0.66)

Table S2. Cohort life table of male dogs under primary veterinary care in the UK.

Age (year) [x, x+1)	Number of dogs died in [x, x+1) (d_x)	Number of dogs living at x (l_x)	Probability of dogs dying in [x, x+1) ($\hat{q}_x$)	Mean fraction of last year of life lived by dogs died in [x, x+1) ($\hat{a}_x$)	Number of dog−years lived in [x, x+1) (L_x)	Number of dogs living at year x (T_x)	Life expectancy at year x ($\hat{e}_x$)
0−1	277	15989	0.017	0.42	15829.72	176988.34	11.07 (11.01−11.13)
1−2	293	15712	0.019	0.52	15571.89	161158.62	10.26 (10.20−10.32)
2−3	272	15419	0.018	0.46	15270.94	145586.73	9.44 (9.39−9.50)
3−4	276	15147	0.018	0.50	15008.65	130315.79	8.60 (8.55−8.66)
4−5	334	14871	0.022	0.46	14691.98	115307.14	7.75 (7.70−7.80)
5−6	379	14537	0.026	0.48	14339.04	100615.17	6.92 (6.87−6.97)
6−7	480	14158	0.034	0.45	13893.69	86276.12	6.09 (6.05−6.14)
7−8	691	13678	0.051	0.51	13338.66	72382.43	5.29 (5.25−5.34)
8−9	950	12987	0.073	0.50	12507.89	59043.77	4.55 (4.50−4.59)
9−10	1214	12037	0.101	0.49	11413.07	46535.89	3.87 (3.83−3.91)
10−11	1501	10823	0.139	0.48	10040.48	35122.81	3.25 (3.21−3.28)
11−12	1800	9322	0.193	0.50	8414.53	25082.34	2.69 (2.65−2.73)
12−13	1864	7522	0.248	0.47	6536.32	16667.81	2.22 (2.18−2.25)
13−14	1909	5658	0.337	0.48	4656.61	10131.49	1.79 (1.76−1.83)
14−15	1548	3749	0.413	0.44	2885.71	5474.88	1.46 (1.42−1.50)
15−16	1146	2201	0.521	0.42	1531.77	2589.18	1.18 (1.13−1.22)
16−17	618	1055	0.586	0.39	679.36	1057.41	1.00 (0.95−1.06)
17−18	287	437	0.657	0.39	260.60	378.05	0.87 (0.79−0.94)
18−19	97	150	0.647	0.30	82.00	117.45	0.78 (0.66−0.91)
19+	53	53	1	0.67	35.45	35.45	0.67 (0.50−0.85)

Table S3. Cohort life table of entire female dogs under primary veterinary care in the UK.

Age (year) [x, x+1)	Number of dogs died in [x, x+1) (d_x)	Number of dogs living at x (l_x)	Probability of dogs dying in [x, x+1) ($\hat{q}_x$)	Mean fraction of last year of life lived by dogs died in [x, x+1) ($\hat{a}_x$)	Number of dog−years lived in [x, x+1) (L_x)	Number of dogs living at year x (T_x)	Life expectancy at year x ($\hat{e}_x$)
0−1	224	5623	0.040	0.39	5486.48	59040.80	10.5 (10.38−10.61)
1−2	145	5399	0.027	0.51	5328.03	53554.32	9.92 (9.81−10.03)
2−3	153	5254	0.029	0.43	5167.34	48226.28	9.18 (9.08−9.28)
3−4	132	5101	0.026	0.42	5024.78	43058.94	8.44 (8.35−8.54)
4−5	123	4969	0.025	0.47	4904.42	38034.16	7.65 (7.57−7.74)
5−6	144	4846	0.030	0.42	4762.33	33129.74	6.84 (6.75−6.92)
6−7	189	4702	0.040	0.49	4604.69	28367.41	6.03 (5.95−6.11)
7−8	246	4513	0.055	0.51	4391.81	23762.72	5.27 (5.19−5.34)
8−9	315	4267	0.074	0.47	4099.82	19370.92	4.54 (4.46−4.61)
9−10	408	3952	0.103	0.50	3747.48	15271.10	3.86 (3.79−3.93)
10−11	507	3544	0.143	0.48	3279.46	11523.62	3.25 (3.18−3.32)
11−12	607	3037	0.200	0.48	2724.25	8244.16	2.71 (2.65−2.78)
12−13	601	2430	0.247	0.44	2094.04	5519.91	2.27 (2.21−2.33)
13−14	568	1829	0.311	0.48	1531.01	3425.87	1.87 (1.81−1.94)
14−15	490	1261	0.389	0.41	970.84	1894.86	1.50 (1.44−1.57)
15−16	393	771	0.510	0.40	535.20	924.02	1.20 (1.12−1.28)
16−17	213	378	0.563	0.36	241.55	388.81	1.03 (0.93−1.13)
17−18	94	165	0.570	0.34	103.17	147.26	0.89 (0.77−1.02)
18−19	49	71	0.690	0.24	33.89	44.09	0.62 (0.47−0.78)
19+	22	22	1	0.46	10.19	10.19	0.46 (0.27−0.66)

Table S4. Cohort life table of neutered female dogs under primary veterinary care in the UK.

Age (year) [x, x+1)	Number of dogs died in [x, x+1) (d_x)	Number of dogs living at x (l_x)	Probability of dogs dying in [x, x+1) ($\hat{q}_x$)	Mean fraction of last year of life lived by dogs died in [x, x+1) ($\hat{a}_x$)	Number of dog−years lived in [x, x+1) (L_x)	Number of dogs living at year x (T_x)	Life expectancy at year x ($\hat{e}_x$)
0−1	13	8951	0.001	0.74	8947.64	107192.25	11.98 (11.91−12.04)
1−2	43	8938	0.005	0.56	8919.26	98244.61	10.99 (10.93−11.06)
2−3	57	8895	0.006	0.50	8866.28	89325.35	10.04 (9.98−10.10)
3−4	81	8838	0.009	0.48	8796.22	80459.07	9.10 (9.04−9.17)
4−5	102	8757	0.012	0.52	8708.33	71662.86	8.18 (8.12−8.24)
5−6	154	8655	0.018	0.50	8578.39	62954.53	7.27 (7.22−7.33)
6−7	223	8501	0.026	0.48	8384.67	54376.14	6.40 (6.34−6.45)
7−8	317	8278	0.038	0.51	8123.24	45991.47	5.56 (5.50−5.61)
8−9	465	7961	0.058	0.52	7736.66	37868.23	4.76 (4.71−4.81)
9−10	643	7496	0.086	0.50	7172.95	30131.57	4.02 (3.97−4.07)
10−11	844	6853	0.123	0.49	6426.73	22958.62	3.35 (3.30−3.40)
11−12	1042	6009	0.173	0.51	5498.80	16531.89	2.75 (2.71−2.79)
12−13	1180	4967	0.238	0.50	4371.14	11033.10	2.22 (2.18−2.26)
13−14	1308	3787	0.345	0.49	3116.36	6661.96	1.76 (1.72−1.80)
14−15	1088	2479	0.439	0.46	1887.09	3545.60	1.43 (1.38−1.48)
15−16	710	1391	0.510	0.45	1003.25	1658.51	1.19 (1.14−1.24)
16−17	422	681	0.620	0.40	427.98	655.26	0.96 (0.90−1.03)
17−18	161	259	0.622	0.38	159.01	227.28	0.88 (0.79−0.97)
18−19	72	98	0.735	0.40	54.82	68.27	0.70 (0.57−0.83)
19+	26	26	1	0.52	13.45	13.45	0.52 (0.31−0.78)

Table S5. Cohort life table of entire male dogs under primary veterinary care in the UK.

Age (year) [x, x+1)	Number of dogs died in [x, x+1) (d_x)	Number of dogs living at x (l_x)	Probability of dogs dying in [x, x+1) ($\hat{q}_x$)	Mean fraction of last year of life lived by dogs died in [x, x+1) ($\hat{a}_x$)	Number of dog−years lived in [x, x+1) (L_x)	Number of dogs living at year x (T_x)	Life expectancy at year x ($\hat{e}_x$)
0−1	260	7394	0.035	0.41	7240.00	78197.63	10.58 (10.48−10.68)
1−2	226	7134	0.032	0.51	7023.68	70957.63	9.95 (9.86−10.04)
2−3	178	6908	0.026	0.46	6811.21	63933.95	9.26 (9.17−9.34)
3−4	167	6730	0.025	0.48	6643.55	57122.74	8.49 (8.41−8.57)
4−5	169	6563	0.026	0.43	6466.80	50479.19	7.69 (7.62−7.77)
5−6	178	6394	0.028	0.46	6297.40	44012.39	6.88 (6.81−6.95)
6−7	234	6216	0.038	0.42	6080.26	37714.99	6.07 (6.00−6.14)
7−8	297	5982	0.050	0.51	5835.51	31634.73	5.29 (5.22−5.35)
8−9	410	5685	0.072	0.48	5471.86	25799.22	4.54 (4.48−4.60)
9−10	550	5275	0.104	0.46	4975.53	20327.36	3.85 (3.79−3.91)
10−11	658	4725	0.139	0.45	4363.43	15351.83	3.25 (3.19−3.31)
11−12	797	4067	0.196	0.49	3660.85	10988.39	2.70 (2.65−2.76)
12−13	792	3270	0.242	0.47	2852.42	7327.54	2.24 (2.19−2.30)
13−14	794	2478	0.320	0.46	2046.17	4475.12	1.81 (1.75−1.86)
14−15	692	1684	0.411	0.43	1290.75	2428.95	1.44 (1.39−1.50)
15−16	528	992	0.532	0.40	674.43	1138.21	1.15 (1.08−1.21)
16−17	261	464	0.562	0.34	291.95	463.78	1.00 (0.91−1.09)
17−18	126	203	0.621	0.33	118.66	171.82	0.85 (0.73−0.97)
18−19	53	77	0.688	0.27	38.29	53.17	0.69 (0.53−0.87)
19+	24	24	1	0.62	14.87	14.87	0.62 (0.38−0.89)

Table S6. Cohort life table of neutered male dogs under primary veterinary care in the UK.

Age (year) [x, x+1)	Number of dogs died in [x, x+1) (d_x)	Number of dogs living at x (l_x)	Probability of dogs dying in [x, x+1) ($\hat{q}_x$)	Mean fraction of last year of life lived by dogs died in [x, x+1) ($\hat{a}_x$)	Number of dog−years lived in [x, x+1) (L_x)	Number of dogs living at year x (T_x)	Life expectancy at year x ($\hat{e}_x$)
0−1	17	8595	0.002	0.69	8589.72	98790.71	11.49 (11.42−11.57)
1−2	67	8578	0.008	0.56	8548.21	90200.99	10.52 (10.44−10.59)
2−3	94	8511	0.011	0.45	8459.72	81652.78	9.59 (9.52−9.66)
3−4	109	8417	0.013	0.52	8365.10	73193.05	8.70 (8.63−8.76)
4−5	165	8308	0.020	0.50	8225.18	64827.96	7.80 (7.74−7.87)
5−6	201	8143	0.025	0.50	8041.64	56602.78	6.95 (6.89−7.01)
6−7	246	7942	0.031	0.48	7813.43	48561.14	6.11 (6.06−6.17)
7−8	394	7696	0.051	0.51	7503.15	40747.70	5.29 (5.24−5.35)
8−9	540	7302	0.074	0.51	7036.02	33244.55	4.55 (4.50−4.61)
9−10	664	6762	0.098	0.51	6437.54	26208.52	3.88 (3.82−3.93)
10−11	843	6098	0.138	0.50	5677.04	19770.99	3.24 (3.19−3.29)
11−12	1003	5255	0.191	0.50	4753.68	14093.94	2.68 (2.63−2.73)
12−13	1072	4252	0.252	0.47	3683.89	9340.27	2.20 (2.15−2.24)
13−14	1115	3180	0.351	0.49	2610.44	5656.37	1.78 (1.73−1.83)
14−15	856	2065	0.415	0.45	1594.96	3045.93	1.48 (1.42−1.53)
15−16	618	1209	0.511	0.43	857.34	1450.97	1.20 (1.14−1.26)
16−17	357	591	0.604	0.43	387.41	593.63	1.00 (0.93−1.08)
17−18	161	234	0.688	0.43	141.94	206.23	0.88 (0.78−0.99)
18−19	44	73	0.603	0.33	43.70	64.29	0.88 (0.70−1.08)
19+	29	29	1	0.71	20.58	20.58	0.71 (0.48−0.96)

Table S7. Cohort life table of Gundogs under primary veterinary care in the UK.

Age (year) [x, x+1)	Number of dogs died in [x, x+1) (d_x)	Number of dogs living at x (l_x)	Probability of dogs dying in [x, x+1) ($\hat{q}_x$)	Mean fraction of last year of life lived by dogs died in [x, x+1) ($\hat{a}_x$)	Number of dog−years lived in [x, x+1) (L_x)	Number of dogs living at year x (T_x)	Life expectancy at year x ($\hat{e}_x$)
0−1	37	5354	0.007	0.47	5334.47	62507.65	11.67 (11.59−11.76)
1−2	50	5317	0.009	0.50	5292.21	57173.19	10.75 (10.67−10.83)
2−3	45	5267	0.009	0.50	5244.47	51880.98	9.85 (9.77−9.92)
3−4	46	5222	0.009	0.54	5200.65	46636.51	8.93 (8.86−9.00)
4−5	59	5176	0.011	0.51	5147.37	41435.85	8.01 (7.94−8.07)
5−6	65	5117	0.013	0.46	5082.09	36288.48	7.09 (7.03−7.16)
6−7	108	5052	0.021	0.44	4991.30	31206.39	6.18 (6.11−6.24)
7−8	168	4944	0.034	0.52	4863.17	26215.09	5.30 (5.24−5.36)
8−9	246	4776	0.052	0.53	4659.55	21351.92	4.47 (4.41−4.53)
9−10	332	4530	0.073	0.51	4367.79	16692.38	3.68 (3.63−3.74)
10−11	526	4198	0.125	0.52	3943.11	12324.58	2.94 (2.89−2.98)
11−12	771	3672	0.210	0.51	3291.60	8381.47	2.28 (2.24−2.33)
12−13	878	2901	0.303	0.48	2444.20	5089.87	1.75 (1.71−1.80)
13−14	894	2023	0.442	0.47	1548.75	2645.67	1.31 (1.27−1.35)
14−15	654	1129	0.579	0.43	759.31	1096.92	0.97 (0.93−1.02)
15−16	353	475	0.743	0.41	266.00	337.60	0.71 (0.65−0.77)
16−17	96	122	0.787	0.29	54.11	71.60	0.59 (0.47−0.72)
17+	26	26	1	0.67	17.49	17.49	0.67 (0.41−0.97)

Table S8. Cohort life table of Hound dogs under primary veterinary care in the UK.

Age (year) [x, x+1)	Number of dogs died in [x, x+1) (d_x)	Number of dogs living at x (l_x)	Probability of dogs dying in [x, x+1) ($\hat{q}_x$)	Mean fraction of last year of life lived by dogs died in [x, x+1) ($\hat{a}_x$)	Number of dog−years lived in [x, x+1) (L_x)	Number of dogs living at year x (T_x)	Life expectancy at year x ($\hat{e}_x$)
0−1	15	1329	0.011	0.46	1320.89	14232.81	10.71 (10.53−10.89)
1−2	14	1314	0.011	0.52	1307.28	12911.93	9.83 (9.66−10.00)
2−3	23	1300	0.018	0.48	1287.96	11604.65	8.93 (8.76−9.09)
3−4	12	1277	0.009	0.50	1271.04	10316.69	8.08 (7.93−8.23)
4−5	29	1265	0.023	0.44	1248.72	9045.66	7.15 (7.00−7.30)
5−6	34	1236	0.028	0.59	1221.91	7796.93	6.31 (6.17−6.45)
6−7	38	1202	0.032	0.40	1179.14	6575.02	5.47 (5.34−5.61)
7−8	65	1164	0.056	0.47	1129.51	5395.88	4.64 (4.51−4.76)
8−9	95	1099	0.086	0.52	1053.24	4266.37	3.88 (3.76−4.00)
9−10	123	1004	0.123	0.50	942.68	3213.13	3.20 (3.09−3.31)
10−11	145	881	0.165	0.50	808.54	2270.45	2.58 (2.48−2.68)
11−12	196	736	0.266	0.53	643.75	1461.90	1.99 (1.89−2.09)
12−13	208	540	0.385	0.46	427.81	818.16	1.52 (1.42−1.62)
13−14	183	332	0.551	0.48	237.06	390.35	1.18 (1.07−1.29)
14−15	92	149	0.617	0.42	95.97	153.28	1.03 (0.88−1.19)
15−16	36	57	0.632	0.44	36.92	57.31	1.01 (0.79−1.25)
16−17	10	21	0.476	0.37	14.71	20.39	0.97 (0.68−1.28)
17+	11	11	1	0.52	5.68	5.68	0.52 (0.27−0.77)

Table S9. Cohort life table of non−Kennel Club recognised dogs under primary veterinary care in the UK.

Age (year) [x, x+1)	Number of dogs died in [x, x+1) (d_x)	Number of dogs living at x (l_x)	Probability of dogs dying in [x, x+1) ($\hat{q}_x$)	Mean fraction of last year of life lived by dogs died in [x, x+1) ($\hat{a}_x$)	Number of dog−years lived in [x, x+1) (L_x)	Number of dogs living at year x (T_x)	Life expectancy at year x ($\hat{e}_x$)
0−1	139	7060	0.020	0.42	6979.92	82328.21	11.66 (11.56−11.76)
1−2	135	6921	0.020	0.52	6856.80	75348.29	10.89 (10.79−10.98)
2−3	112	6786	0.017	0.42	6720.90	68491.49	10.09 (10.01−10.18)
3−4	134	6674	0.020	0.42	6596.92	61770.59	9.26 (9.17−9.34)
4−5	135	6540	0.021	0.48	6469.76	55173.67	8.44 (8.36−8.52)
5−6	163	6405	0.025	0.45	6315.36	48703.90	7.60 (7.53−7.68)
6−7	177	6242	0.028	0.48	6150.65	42388.55	6.79 (6.72−6.86)
7−8	234	6065	0.039	0.51	5950.47	36237.90	5.97 (5.91−6.04)
8−9	336	5831	0.058	0.48	5656.91	30287.43	5.19 (5.13−5.26)
9−10	407	5495	0.074	0.50	5291.39	24630.52	4.48 (4.42−4.54)
10−11	520	5088	0.102	0.48	4815.37	19339.13	3.80 (3.74−3.86)
11−12	625	4568	0.137	0.49	4250.00	14523.76	3.18 (3.13−3.23)
12−13	700	3943	0.178	0.47	3575.05	10273.76	2.61 (2.55−2.66)
13−14	874	3243	0.270	0.49	2794.18	6698.71	2.07 (2.02−2.12)
14−15	822	2369	0.347	0.45	1920.26	3904.53	1.65 (1.60−1.70)
15−16	733	1547	0.474	0.43	1130.62	1984.27	1.28 (1.23−1.34)
16−17	458	814	0.563	0.40	540.35	853.65	1.05 (0.98−1.11)
17−18	218	356	0.612	0.37	217.74	313.31	0.88 (0.80−0.96)
18−19	99	138	0.717	0.35	73.43	95.56	0.69 (0.58−0.82)
19+	39	39	1	0.57	22.14	22.14	0.57 (0.37−0.80)

Table S10. Cohort life table of Pastoral dogs under primary veterinary care in the UK.

Age (year) [x, x+1)	Number of dogs died in [x, x+1) (d_x)	Number of dogs living at x (l_x)	Probability of dogs dying in [x, x+1) ($\hat{q}_x$)	Mean fraction of last year of life lived by dogs died in [x, x+1) ($\hat{a}_x$)	Number of dog−years lived in [x, x+1) (L_x)	Number of dogs living at year x (T_x)	Life expectancy at year x ($\hat{e}_x$)
0−1	32	2451	0.013	0.52	2435.76	27458.54	11.20 (11.06−11.35)
1−2	44	2419	0.018	0.51	2397.29	25022.79	10.34 (10.21−10.48)
2−3	44	2375	0.019	0.45	2350.73	22625.50	9.53 (9.40−9.66)
3−4	24	2331	0.010	0.49	2318.75	20274.77	8.70 (8.58−8.82)
4−5	34	2307	0.015	0.50	2290.16	17956.02	7.78 (7.67−7.90)
5−6	38	2273	0.017	0.41	2250.49	15665.86	6.89 (6.78−7.00)
6−7	69	2235	0.031	0.49	2199.59	13415.37	6.00 (5.90−6.11)
7−8	91	2166	0.042	0.50	2120.61	11215.78	5.18 (5.08−5.28)
8−9	127	2075	0.061	0.51	2012.18	9095.17	4.38 (4.29−4.48)
9−10	231	1948	0.119	0.50	1832.09	7083.00	3.64 (3.54−3.73)
10−11	235	1717	0.137	0.50	1599.31	5250.90	3.06 (2.97−3.14)
11−12	322	1482	0.217	0.51	1324.9	3651.59	2.46 (2.38−2.55)
12−13	322	1160	0.278	0.49	995.99	2326.69	2.01 (1.93−2.09)
13−14	318	838	0.379	0.47	668.57	1330.70	1.59 (1.51−1.67)
14−15	241	520	0.463	0.44	384.80	662.13	1.27 (1.19−1.36)
15−16	158	279	0.566	0.41	186.13	277.33	0.99 (0.89−1.10)
16−17	86	121	0.711	0.36	66.18	91.20	0.75 (0.62−0.90)
17+	35	35	1	0.71	25.02	25.02	0.71 (0.49−0.98)

Table S11. Cohort life table of Terriers under primary veterinary care in the UK.

Age (year) [x, x+1)	Number of dogs died in [x, x+1) (d_x)	Number of dogs living at x (l_x)	Probability of dogs dying in [x, x+1) ($\hat{q}_x$)	Mean fraction of last year of life lived by dogs died in [x, x+1) ($\hat{a}_x$)	Number of dog−years lived in [x, x+1) (L_x)	Number of dogs living at year x (T_x)	Life expectancy at year x ($\hat{e}_x$)
0−1	51	6055	0.008	0.44	6026.52	72824.66	12.03 (11.94−12.12)
1−2	60	6004	0.010	0.54	5976.29	66798.13	11.13 (11.04−11.21)
2−3	59	5944	0.010	0.49	5913.97	60821.84	10.23 (10.15−10.32)
3−4	72	5885	0.012	0.49	5848.46	54907.86	9.33 (9.25−9.41)
4−5	76	5813	0.013	0.48	5773.29	49059.40	8.44 (8.36−8.52)
5−6	101	5737	0.018	0.50	5686.14	43286.11	7.55 (7.47−7.62)
6−7	141	5636	0.025	0.46	5560.23	37599.97	6.67 (6.60−6.74)
7−8	203	5495	0.037	0.49	5390.67	32039.74	5.83 (5.76−5.90)
8−9	254	5292	0.048	0.48	5159.08	26649.06	5.04 (4.97−5.10)
9−10	369	5038	0.073	0.46	4838.05	21489.99	4.27 (4.20−4.33)
10−11	559	4669	0.120	0.48	4380.26	16651.94	3.57 (3.51−3.63)
11−12	612	4110	0.149	0.50	3805.00	12271.67	2.99 (2.93−3.04)
12−13	739	3498	0.211	0.48	3114.97	8466.68	2.42 (2.37−2.47)
13−14	813	2759	0.295	0.50	2348.65	5351.71	1.94 (1.89−1.99)
14−15	756	1946	0.388	0.45	1528.27	3003.06	1.54 (1.49−1.60)
15−16	580	1190	0.487	0.42	852.24	1474.79	1.24 (1.18−1.30)
16−17	358	610	0.587	0.40	395.63	622.55	1.02 (0.95−1.10)
17−18	158	252	0.627	0.40	156.47	226.92	0.90 (0.80−1.01)
18−19	61	94	0.649	0.30	51.21	70.46	0.75 (0.61−0.90)
19+	33	33	1	0.58	19.25	19.25	0.58 (0.39−0.80)

Table S12. Cohort life table of Toy dogs under primary veterinary care in the UK.

Age (year) [x, x+1)	Number of dogs died in [x, x+1) (d_x)	Number of dogs living at x (l_x)	Probability of dogs dying in [x, x+1) ($\hat{q}_x$)	Mean fraction of last year of life lived by dogs died in [x, x+1) ($\hat{a}_x$)	Number of dog−years lived in [x, x+1) (L_x)	Number of dogs living at year x (T_x)	Life expectancy at year x ($\hat{e}_x$)
0−1	116	3334	0.035	0.37	3260.48	35584.72	10.67 (10.54−10.81)
1−2	58	3218	0.018	0.50	3189.17	32324.24	10.04 (9.92−10.17)
2−3	66	3160	0.021	0.38	3119.15	29135.07	9.22 (9.10−9.34)
3−4	55	3094	0.018	0.47	3064.71	26015.92	8.41 (8.29−8.52)
4−5	70	3039	0.023	0.54	3006.79	22951.20	7.55 (7.44−7.66)
5−6	77	2969	0.026	0.45	2926.35	19944.42	6.72 (6.61−6.82)
6−7	106	2892	0.037	0.44	2832.80	17018.06	5.88 (5.78−5.99)
7−8	154	2786	0.055	0.53	2713.61	14185.27	5.09 (5.00−5.19)
8−9	232	2632	0.088	0.52	2519.94	11471.66	4.36 (4.27−4.45)
9−10	296	2400	0.123	0.52	2256.66	8951.71	3.73 (3.64−3.82)
10−11	322	2104	0.153	0.46	1930.59	6695.05	3.18 (3.09−3.27)
11−12	386	1782	0.217	0.50	1588.84	4764.45	2.67 (2.59−2.76)
12−13	343	1396	0.246	0.48	1216.65	3175.61	2.27 (2.19−2.36)
13−14	347	1053	0.330	0.47	870.15	1958.96	1.86 (1.78−1.95)
14−15	289	706	0.409	0.43	540.99	1088.81	1.54 (1.45−1.64)
15−16	187	417	0.448	0.42	309.15	547.82	1.31 (1.21−1.42)
16−17	131	230	0.570	0.40	151.28	238.66	1.04 (0.92−1.16)
17−18	63	99	0.636	0.38	60.10	87.38	0.88 (0.72−1.05)
18−19	25	36	0.694	0.36	20.06	27.29	0.76 (0.54−0.99)
19+	11	11	1	0.66	7.23	7.23	0.66 (0.44−0.87)

Table S13. Cohort life table of Utility dogs under primary veterinary care in the UK.

Age (year) [x, x+1)	Number of dogs died in [x, x+1) (d_x)	Number of dogs living at x (l_x)	Probability of dogs dying in [x, x+1) ($\hat{q}_x$)	Mean fraction of last year of life lived by dogs died in [x, x+1) ($\hat{a}_x$)	Number of dog−years lived in [x, x+1) (L_x)	Number of dogs living at year x (T_x)	Life expectancy at year x ($\hat{e}_x$)
0−1	94	2707	0.035	0.38	2648.93	27225.49	10.06 (9.89−10.23)
1−2	76	2613	0.029	0.55	2578.47	24576.56	9.41 (9.25−9.57)
2−3	98	2537	0.039	0.53	2490.75	21998.08	8.67 (8.52−8.82)
3−4	92	2439	0.038	0.48	2390.95	19507.33	8.00 (7.85−8.14)
4−5	90	2347	0.038	0.46	2298.62	17116.39	7.29 (7.16−7.43)
5−6	106	2257	0.047	0.48	2201.46	14817.77	6.57 (6.44−6.70)
6−7	105	2151	0.049	0.44	2092.60	12616.31	5.87 (5.74−5.99)
7−8	135	2046	0.066	0.51	1980.34	10523.71	5.14 (5.03−5.26)
8−9	192	1911	0.100	0.47	1810.07	8543.37	4.47 (4.36−4.58)
9−10	182	1719	0.106	0.48	1624.76	6733.31	3.92 (3.81−4.03)
10−11	219	1537	0.142	0.48	1423.91	5108.55	3.32 (3.22−3.43)
11−12	233	1318	0.177	0.46	1192.91	3684.64	2.80 (2.70−2.89)
12−13	276	1085	0.254	0.46	934.73	2491.72	2.30 (2.20−2.39)
13−14	240	809	0.297	0.49	687.13	1556.99	1.92 (1.83−2.02)
14−15	222	569	0.390	0.43	442.75	869.86	1.53 (1.43−1.63)
15−16	173	347	0.499	0.46	254.06	427.11	1.23 (1.12−1.34)
16−17	105	174	0.603	0.37	107.69	173.04	0.99 (0.85−1.14)
17−18	42	69	0.609	0.40	43.88	65.35	0.95 (0.75−1.17)
18+	27	27	1	0.80	21.47	21.47	0.80 (0.50−1.14)

Table S14. Cohort life table of Working dogs under primary veterinary care in the UK.

Age (year) [x, x+1)	Number of dogs died in [x, x+1) (d_x)	Number of dogs living at x (l_x)	Probability of dogs dying in [x, x+1) ($\hat{q}_x$)	Mean fraction of last year of life lived by dogs died in [x, x+1) ($\hat{a}_x$)	Number of dog−years lived in [x, x+1) (L_x)	Number of dogs living at year x (T_x)	Life expectancy at year x ($\hat{e}_x$)
0−1	30	2184	0.014	0.46	2167.88	19963.70	9.14 (9.01−9.27)
1−2	43	2154	0.020	0.52	2133.16	17795.82	8.26 (8.14−8.38)
2−3	34	2111	0.016	0.37	2089.42	15662.66	7.42 (7.30−7.53)
3−4	52	2077	0.025	0.51	2051.30	13573.24	6.54 (6.42−6.65)
4−5	63	2025	0.031	0.39	1986.45	11521.94	5.69 (5.58−5.80)
5−6	91	1962	0.046	0.49	1915.61	9535.49	4.86 (4.76−4.96)
6−7	145	1871	0.077	0.51	1799.23	7619.87	4.07 (3.98−4.17)
7−8	204	1726	0.118	0.52	1628.33	5820.65	3.37 (3.28−3.46)
8−9	243	1522	0.160	0.50	1399.99	4192.32	2.75 (2.67−2.84)
9−10	318	1279	0.249	0.47	1111.74	2792.33	2.18 (2.10−2.27)
10−11	320	961	0.333	0.44	783.39	1680.59	1.75 (1.66−1.83)
11−12	299	641	0.466	0.48	484.37	897.20	1.40 (1.31−1.49)
12−13	174	342	0.509	0.42	241.54	412.84	1.21 (1.09−1.33)
13−14	101	168	0.601	0.40	107.68	171.29	1.02 (0.87−1.17)
14−15	40	67	0.597	0.35	40.98	63.62	0.95 (0.73−1.19)
15+	27	27	1	0.84	22.63	22.63	0.84 (0.51−1.20)

Table S15. Cohort life table of American Bulldogs under primary veterinary care in the UK.

Age (year) [x, x+1)	Number of dogs died in [x, x+1) (d_x)	Number of dogs living at x (l_x)	Probability of dogs dying in [x, x+1) ($\hat{q}_x$)	Mean fraction of last year of life lived by dogs died in [x, x+1) ($\hat{a}_x$)	Number of dog−years lived in [x, x+1) (L_x)	Number of dogs living at year x (T_x)	Life expectancy at year x ($\hat{e}_x$)
0−1	3	126	0.024	0.37	124.10	988.41	7.84 (7.16−8.33)
1−2	8	123	0.065	0.60	119.79	864.31	7.03 (6.40−7.59)
2−3	5	115	0.043	0.55	112.73	744.53	6.47 (5.88−6.98)
3−4	8	110	0.073	0.53	106.25	631.79	5.74 (5.19−6.26)
4−5	8	102	0.078	0.49	97.91	525.54	5.15 (4.65−5.64)
5−6	6	94	0.064	0.36	90.15	427.63	4.55 (4.07−5.02)
6−7	10	88	0.114	0.44	82.39	337.48	3.83 (3.38−4.30)
7−8	10	78	0.128	0.58	73.77	255.09	3.27 (2.85−3.70)
8−9	12	68	0.176	0.48	61.72	181.32	2.67 (2.27−3.10)
9−10	18	56	0.321	0.60	48.80	119.60	2.14 (1.73−2.55)
10−11	12	38	0.316	0.52	32.26	70.81	1.86 (1.43−2.33)
11−12	13	26	0.500	0.48	19.24	38.55	1.48 (1.01−1.99)
12+	13	13	1	1.48	19.30	19.30	1.48 (0.95−2.03)

Table S16. Cohort life table of Beagles under primary veterinary care in the UK.

Age (year) [x, x+1)	Number of dogs died in [x, x+1) (d_x)	Number of dogs living at x (l_x)	Probability of dogs dying in [x, x+1) ($\hat{q}_x$)	Mean fraction of last year of life lived by dogs died in [x, x+1) ($\hat{a}_x$)	Number of dog−years lived in [x, x+1) (L_x)	Number of dogs living at year x (T_x)	Life expectancy at year x ($\hat{e}_x$)
0−1	5	171	0.029	0.34	167.72	1683.49	9.84 (9.17−10.24)
1−2	3	166	0.018	0.59	164.78	1515.77	9.13 (8.50−9.52)
2−3	6	163	0.037	0.57	160.44	1350.99	8.29 (7.72−8.73)
3−4	6	157	0.038	0.76	155.58	1190.55	7.58 (7.06−8.01)
4−5	7	151	0.046	0.41	146.90	1034.97	6.85 (6.36−7.27)
5−6	4	144	0.028	0.46	141.83	888.06	6.17 (5.72−6.56)
6−7	8	140	0.057	0.53	136.24	746.23	5.33 (4.92−5.75)
7−8	8	132	0.061	0.39	127.10	609.99	4.62 (4.24−5.01)
8−9	12	124	0.097	0.61	119.36	482.89	3.89 (3.55−4.28)
9−10	12	112	0.107	0.42	105.10	363.52	3.25 (2.91−3.60)
10−11	19	100	0.190	0.50	90.54	258.43	2.58 (2.27−2.92)
11−12	22	81	0.272	0.58	71.71	167.88	2.07 (1.77−2.38)
12−13	19	59	0.322	0.50	49.59	96.17	1.63 (1.34−1.95)
13−14	22	40	0.550	0.41	27.12	46.57	1.16 (0.84−1.54)
14+	18	18	1	1.08	19.45	19.45	1.08 (0.62−1.59)

Table S17. Cohort life table of Border Collies under primary veterinary care in the UK.

Age (year) [x, x+1)	Number of dogs died in [x, x+1) (d_x)	Number of dogs living at x (l_x)	Probability of dogs dying in [x, x+1) ($\hat{q}_x$)	Mean fraction of last year of life lived by dogs died in [x, x+1) ($\hat{a}_x$)	Number of dog−years lived in [x, x+1) (L_x)	Number of dogs living at year x (T_x)	Life expectancy at year x ($\hat{e}_x$)
0−1	11	938	0.012	0.50	932.48	11344.05	12.09 (11.85−12.33)
1−2	19	927	0.020	0.50	917.58	10411.58	11.23 (11.00−11.46)
2−3	17	908	0.019	0.39	897.55	9493.99	10.46 (10.24−10.66)
3−4	9	891	0.010	0.46	886.18	8596.44	9.65 (9.45−9.84)
4−5	15	882	0.017	0.42	873.29	7710.27	8.74 (8.56−8.93)
5−6	13	867	0.015	0.41	859.29	6836.98	7.89 (7.71−8.06)
6−7	17	854	0.020	0.50	845.49	5977.69	7.00 (6.83−7.17)
7−8	20	837	0.024	0.45	826.00	5132.20	6.13 (5.98−6.29)
8−9	26	817	0.032	0.51	804.19	4306.20	5.27 (5.12−5.42)
9−10	50	791	0.063	0.47	764.72	3502.01	4.43 (4.29−4.57)
10−11	61	741	0.082	0.51	711.08	2737.29	3.69 (3.57−3.82)
11−12	88	680	0.129	0.56	641.28	2026.22	2.98 (2.86−3.10)
12−13	116	592	0.196	0.51	535.62	1384.93	2.34 (2.23−2.45)
13−14	148	476	0.311	0.50	402.01	849.31	1.78 (1.68−1.89)
14−15	138	328	0.421	0.46	252.80	447.31	1.36 (1.26−1.48)
15−16	101	190	0.532	0.44	133.54	194.51	1.02 (0.91−1.14)
16−17	66	89	0.742	0.34	45.39	60.97	0.69 (0.54−0.84)
17+	23	23	1	0.68	15.58	15.58	0.68 (0.42−1.00)

Table S18. Cohort life table of Boxers under primary veterinary care in the UK.

Age (year) [x, x+1)	Number of dogs died in [x, x+1) (d_x)	Number of dogs living at x (l_x)	Probability of dogs dying in [x, x+1) ($\hat{q}_x$)	Mean fraction of last year of life lived by dogs died in [x, x+1) ($\hat{a}_x$)	Number of dog−years lived in [x, x+1) (L_x)	Number of dogs living at year x (T_x)	Life expectancy at year x ($\hat{e}_x$)
0−1	4	831	0.005	0.07	827.28	8343.83	10.04 (9.85−10.21)
1−2	13	827	0.016	0.57	821.43	7516.55	9.09 (8.91−9.26)
2−3	5	814	0.006	0.40	811.00	6695.12	8.22 (8.06−8.39)
3−4	10	809	0.012	0.49	803.87	5884.11	7.27 (7.11−7.44)
4−5	13	799	0.016	0.34	790.36	5080.25	6.36 (6.21−6.51)
5−6	20	786	0.025	0.51	776.16	4289.89	5.46 (5.31−5.61)
6−7	32	766	0.042	0.52	750.6	3513.73	4.59 (4.45−4.73)
7−8	56	734	0.076	0.51	706.74	2763.13	3.76 (3.63−3.90)
8−9	63	678	0.093	0.56	650.25	2056.39	3.03 (2.91−3.16)
9−10	139	615	0.226	0.48	542.03	1406.14	2.29 (2.17−2.41)
10−11	139	476	0.292	0.48	403.37	864.11	1.82 (1.70−1.93)
11−12	153	337	0.454	0.46	253.75	460.74	1.37 (1.25−1.49)
12−13	101	184	0.549	0.45	128.00	206.99	1.12 (0.98−1.28)
13−14	51	83	0.614	0.35	50.02	78.99	0.95 (0.75−1.18)
14−15	21	32	0.656	0.42	19.80	28.97	0.91 (0.62−1.26)
15+	11	11	1	0.83	9.16	9.16	0.83 (0.29−1.46)

Table S19. Cohort life table of Bulldogs under primary veterinary care in the UK.

Age (year) [x, x+1)	Number of dogs died in [x, x+1) (d_x)	Number of dogs living at x (l_x)	Probability of dogs dying in [x, x+1) ($\hat{q}_x$)	Mean fraction of last year of life lived by dogs died in [x, x+1) ($\hat{a}_x$)	Number of dog−years lived in [x, x+1) (L_x)	Number of dogs living at year x (T_x)	Life expectancy at year x ($\hat{e}_x$)
0−1	19	476	0.040	0.37	464.12	3518.15	7.39 (7.08−7.69)
1−2	25	457	0.055	0.56	446.05	3054.03	6.68 (6.39−6.97)
2−3	26	432	0.060	0.46	418.00	2607.98	6.04 (5.76−6.31)
3−4	23	406	0.057	0.46	393.57	2189.98	5.39 (5.15−5.64)
4−5	27	383	0.070	0.43	367.71	1796.42	4.69 (4.46−4.92)
5−6	31	356	0.087	0.46	339.40	1428.70	4.01 (3.80−4.23)
6−7	30	325	0.092	0.45	308.45	1089.31	3.35 (3.15−3.56)
7−8	48	295	0.163	0.53	272.21	780.86	2.65 (2.46−2.84)
8−9	77	247	0.312	0.44	203.65	508.65	2.06 (1.87−2.25)
9−10	56	170	0.329	0.48	140.66	304.99	1.79 (1.60−1.99)
10−11	46	114	0.404	0.43	87.90	164.34	1.44 (1.25−1.65)
11−12	31	68	0.456	0.40	49.35	76.44	1.12 (0.92−1.34)
12−13	23	37	0.622	0.21	18.90	27.09	0.73 (0.50−0.98)
13+	14	14	1	0.59	8.19	8.19	0.59 (0.35−0.82)

Table S20. Cohort life table of Cavalier King Charles Spaniels under primary veterinary care in the UK.

Age (year) [x, x+1)	Number of dogs died in [x, x+1) (d_x)	Number of dogs living at x (l_x)	Probability of dogs dying in [x, x+1) ($\hat{q}_x$)	Mean fraction of last year of life lived by dogs died in [x, x+1) ($\hat{a}_x$)	Number of dog−years lived in [x, x+1) (L_x)	Number of dogs living at year x (T_x)	Life expectancy at year x ($\hat{e}_x$)
0−1	8	861	0.009	0.31	855.45	9002.55	10.46 (10.26−10.62)
1−2	3	853	0.004	0.80	852.40	8147.10	9.55 (9.36−9.70)
2−3	4	850	0.005	0.85	849.38	7294.71	8.58 (8.41−8.74)
3−4	7	846	0.008	0.44	842.10	6445.33	7.62 (7.45−7.79)
4−5	9	839	0.011	0.57	835.09	5603.23	6.68 (6.52−6.84)
5−6	16	830	0.019	0.55	822.85	4768.13	5.74 (5.59−5.91)
6−7	38	814	0.047	0.49	794.60	3945.28	4.85 (4.69−5.00)
7−8	59	776	0.076	0.56	749.95	3150.68	4.06 (3.91−4.21)
8−9	87	717	0.121	0.55	678.15	2400.73	3.35 (3.21−3.48)
9−10	108	630	0.171	0.50	575.53	1722.58	2.73 (2.60−2.87)
10−11	124	522	0.238	0.46	454.82	1147.05	2.20 (2.07−2.33)
11−12	140	398	0.352	0.48	325.20	692.22	1.74 (1.61−1.87)
12−13	98	258	0.380	0.42	200.88	367.02	1.42 (1.29−1.55)
13−14	89	160	0.556	0.42	108.16	166.15	1.04 (0.90−1.19)
14−15	49	71	0.69	0.39	41.02	57.99	0.82 (0.64−1.01)
15+	22	22	1	0.77	16.97	16.97	0.77 (0.48−1.10)

Table S21. Cohort life table of Chihuahuas under primary veterinary care in the UK.

Age (year) [x, x+1)	Number of dogs died in [x, x+1) (d_x)	Number of dogs living at x (l_x)	Probability of dogs dying in [x, x+1) ($\hat{q}_x$)	Mean fraction of last year of life lived by dogs died in [x, x+1) ($\hat{a}_x$)	Number of dog−years lived in [x, x+1) (L_x)	Number of dogs living at year x (T_x)	Life expectancy at year x ($\hat{e}_x$)
0−1	47	453	0.104	0.39	424.11	3590.88	7.93 (7.48−8.39)
1−2	30	406	0.074	0.48	390.53	3166.77	7.80 (7.37−8.24)
2−3	26	376	0.069	0.34	358.81	2776.25	7.38 (6.97−7.80)
3−4	18	350	0.051	0.42	339.55	2417.44	6.91 (6.52−7.30)
4−5	23	332	0.069	0.46	319.63	2077.89	6.26 (5.88−6.63)
5−6	17	309	0.055	0.45	299.70	1758.26	5.69 (5.34−6.05)
6−7	24	292	0.082	0.36	276.69	1458.56	5.00 (4.64−5.35)
7−8	29	268	0.108	0.46	252.29	1181.87	4.41 (4.07−4.75)
8−9	31	239	0.130	0.49	223.34	929.58	3.89 (3.56−4.22)
9−10	34	208	0.163	0.50	190.88	706.24	3.40 (3.07−3.72)
10−11	38	174	0.218	0.50	155.06	515.36	2.96 (2.64−3.29)
11−12	31	136	0.228	0.41	117.64	360.29	2.65 (2.32−2.99)
12−13	31	105	0.295	0.50	89.39	242.66	2.31 (1.98−2.67)
13−14	25	74	0.338	0.45	60.29	153.27	2.07 (1.71−2.45)
14−15	16	49	0.327	0.53	41.43	92.98	1.90 (1.53−2.29)
15−16	11	33	0.333	0.56	28.12	51.55	1.56 (1.20−1.95)
16+	22	22	1	1.07	23.43	23.43	1.07 (0.67−1.52)

Table S22. Cohort life table of Cocker Spaniels under primary veterinary care in the UK.

Age (year) [x, x+1)	Number of dogs died in [x, x+1) (d_x)	Number of dogs living at x (l_x)	Probability of dogs dying in [x, x+1) ($\hat{q}_x$)	Mean fraction of last year of life lived by dogs died in [x, x+1) ($\hat{a}_x$)	Number of dog−years lived in [x, x+1) (L_x)	Number of dogs living at year x (T_x)	Life expectancy at year x ($\hat{e}_x$)
0−1	13	1063	0.012	0.33	1054.25	12048.30	11.33 (11.13−11.53)
1−2	14	1050	0.013	0.56	1043.88	10994.04	10.47 (10.28−10.65)
2−3	8	1036	0.008	0.54	1032.30	9950.17	9.60 (9.43−9.78)
3−4	13	1028	0.013	0.56	1022.22	8917.86	8.67 (8.51−8.85)
4−5	18	1015	0.018	0.49	1005.78	7895.64	7.78 (7.62−7.95)
5−6	20	997	0.020	0.44	985.85	6889.86	6.91 (6.76−7.07)
6−7	22	977	0.023	0.41	964.09	5904.01	6.04 (5.90−6.19)
7−8	39	955	0.041	0.41	932.09	4939.91	5.17 (5.04−5.31)
8−9	53	916	0.058	0.51	890.14	4007.83	4.38 (4.25−4.51)
9−10	74	863	0.086	0.54	829.07	3117.69	3.61 (3.49−3.74)
10−11	98	789	0.124	0.49	738.56	2288.62	2.90 (2.79−3.02)
11−12	167	691	0.242	0.50	607.02	1550.06	2.24 (2.14−2.35)
12−13	156	524	0.298	0.46	440.09	943.04	1.80 (1.70−1.90)
13−14	145	368	0.394	0.49	294.24	502.95	1.37 (1.27−1.46)
14−15	133	223	0.596	0.44	148.48	208.71	0.94 (0.84−1.03)
15−16	72	90	0.800	0.46	51.01	60.23	0.67 (0.56−0.78)
16+	18	18	1	0.51	9.22	9.22	0.51 (0.33−0.70)

Table S23. Cohort life table of Crossbreeds under primary veterinary care in the UK.

Age (year) [x, x+1)	Number of dogs died in [x, x+1) (d_x)	Number of dogs living at x (l_x)	Probability of dogs dying in [x, x+1) ($\hat{q}_x$)	Mean fraction of last year of life lived by dogs died in [x, x+1) ($\hat{a}_x$)	Number of dog−years lived in [x, x+1) (L_x)	Number of dogs living at year x (T_x)	Life expectancy at year x ($\hat{e}_x$)
0−1	126	6511	0.019	0.43	6439.03	76933.20	11.82 (11.72−11.92)
1−2	113	6385	0.018	0.51	6329.99	70494.18	11.04 (10.95−11.13)
2−3	96	6272	0.015	0.42	6216.08	64164.19	10.23 (10.14−10.32)
3−4	108	6176	0.017	0.41	6112.45	57948.11	9.38 (9.30−9.47)
4−5	119	6068	0.020	0.48	6006.21	51835.66	8.54 (8.46−8.63)
5−6	142	5949	0.024	0.46	5871.75	45829.45	7.70 (7.63−7.78)
6−7	147	5807	0.025	0.49	5732.08	39957.70	6.88 (6.81−6.95)
7−8	207	5660	0.037	0.51	5558.40	34225.62	6.05 (5.98−6.12)
8−9	290	5453	0.053	0.48	5303.38	28667.22	5.26 (5.19−5.33)
9−10	360	5163	0.070	0.49	4979.49	23363.84	4.53 (4.46−4.59)
10−11	477	4803	0.099	0.48	4553.44	18384.35	3.83 (3.77−3.89)
11−12	572	4326	0.132	0.50	4037.19	13830.91	3.20 (3.14−3.25)
12−13	666	3754	0.177	0.47	3400.38	9793.72	2.61 (2.56−2.66)
13−14	820	3088	0.266	0.49	2667.16	6393.34	2.07 (2.02−2.12)
14−15	792	2268	0.349	0.45	1835.28	3726.18	1.64 (1.59−1.69)
15−16	699	1476	0.474	0.43	1076.64	1890.9	1.28 (1.23−1.33)
16−17	438	777	0.564	0.41	516.41	814.26	1.05 (0.98−1.11)
17−18	206	339	0.608	0.37	208.30	297.85	0.88 (0.80−0.96)
18−19	97	133	0.729	0.35	69.94	89.54	0.67 (0.56−0.79)
19+	36	36	1	0.54	19.60	19.60	0.54 (0.36−0.76)

Table S24. Cohort life table of French Bulldogs under primary veterinary care in the UK.

Age (year) [x, x+1)	Number of dogs died in [x, x+1) (d_x)	Number of dogs living at x (l_x)	Probability of dogs dying in [x, x+1) ($\hat{q}_x$)	Mean fraction of last year of life lived by dogs died in [x, x+1) ($\hat{a}_x$)	Number of dog−years lived in [x, x+1) (L_x)	Number of dogs living at year x (T_x)	Life expectancy at year x ($\hat{e}_x$)
0−1	30	229	0.131	0.34	209.06	1041.55	4.55 (4.14−5.01)
1−2	27	199	0.136	0.54	186.64	832.49	4.18 (3.78−4.66)
2−3	35	172	0.203	0.59	157.79	645.85	3.75 (3.33−4.23)
3−4	33	137	0.241	0.56	122.4	488.06	3.56 (3.11−4.07)
4−5	18	104	0.173	0.60	96.80	365.66	3.52 (3.04−4.06)
5−6	18	86	0.209	0.47	76.51	268.87	3.13 (2.62−3.70)
6−7	24	68	0.353	0.37	52.77	192.35	2.83 (2.26−3.43)
7−8	6	44	0.136	0.27	39.64	139.59	3.17 (2.56−3.73)
8−9	11	38	0.289	0.49	32.36	99.95	2.63 (2.04−3.19)
9−10	4	27	0.148	0.28	24.13	67.59	2.50 (1.89−2.95)
10−11	6	23	0.261	0.49	19.91	43.46	1.89 (1.36−2.40)
11+	17	17	1	1.39	23.55	23.55	1.39 (0.84−1.98)

Table S25. Cohort life table of German Shepherd Dogs under primary veterinary care in the UK.

Age (year) [x, x+1)	Number of dogs died in [x, x+1) (d_x)	Number of dogs living at x (l_x)	Probability of dogs dying in [x, x+1) ($\hat{q}_x$)	Mean fraction of last year of life lived by dogs died in [x, x+1) ($\hat{a}_x$)	Number of dog−years lived in [x, x+1) (L_x)	Number of dogs living at year x (T_x)	Life expectancy at year x ($\hat{e}_x$)
0−1	14	1097	0.013	0.53	1090.48	11173.75	10.19 (10−10.37)
1−2	22	1083	0.020	0.51	1072.26	10083.27	9.31 (9.13−9.49)
2−3	20	1061	0.019	0.51	1051.22	9011.01	8.49 (8.33−8.66)
3−4	13	1041	0.012	0.52	1034.80	7959.79	7.65 (7.49−7.80)
4−5	14	1028	0.014	0.61	1022.58	6925.00	6.74 (6.59−6.89)
5−6	23	1014	0.023	0.38	999.71	5902.41	5.82 (5.68−5.97)
6−7	43	991	0.043	0.47	968.09	4902.70	4.95 (4.81−5.09)
7−8	62	948	0.065	0.52	917.94	3934.61	4.15 (4.02−4.28)
8−9	87	886	0.098	0.50	842.55	3016.68	3.40 (3.28−3.53)
9−10	150	799	0.188	0.51	724.77	2174.13	2.72 (2.61−2.84)
10−11	134	649	0.206	0.48	579.79	1449.36	2.23 (2.12−2.35)
11−12	189	515	0.367	0.47	415.72	869.57	1.69 (1.58−1.80)
12−13	142	326	0.436	0.46	249.80	453.86	1.39 (1.27−1.52)
13−14	100	184	0.543	0.42	126.39	204.05	1.11 (0.97−1.26)
14−15	52	84	0.619	0.39	52.54	77.66	0.92 (0.74−1.13)
15+	32	32	1	0.79	25.13	25.13	0.79 (0.50−1.14)

Table S26. Cohort life table of Huskies under primary veterinary care in the UK.

Age (year) [x, x+1)	Number of dogs died in [x, x+1) (d_x)	Number of dogs living at x (l_x)	Probability of dogs dying in [x, x+1) ($\hat{q}_x$)	Mean fraction of last year of life lived by dogs died in [x, x+1) ($\hat{a}_x$)	Number of dog−years lived in [x, x+1) (L_x)	Number of dogs living at year x (T_x)	Life expectancy at year x ($\hat{e}_x$)
0−1	4	153	0.026	0.40	150.62	1453.90	9.50 (8.71−9.88)
1−2	7	149	0.047	0.43	144.98	1303.28	8.75 (8.04−9.15)
2−3	4	142	0.028	0.31	139.24	1158.30	8.16 (7.51−8.51)
3−4	4	138	0.029	0.35	135.39	1019.06	7.38 (6.78−7.76)
4−5	4	134	0.030	0.41	131.66	883.67	6.59 (6.05−6.99)
5−6	4	130	0.031	0.27	127.08	752.02	5.78 (5.30−6.20)
6−7	8	126	0.063	0.39	121.12	624.94	4.96 (4.51−5.41)
7−8	14	118	0.119	0.34	108.71	503.82	4.27 (3.85−4.70)
8−9	5	104	0.048	0.62	102.09	395.11	3.80 (3.41−4.17)
9−10	16	99	0.162	0.52	91.25	293.02	2.96 (2.59−3.34)
10−11	21	83	0.253	0.46	71.60	201.77	2.43 (2.07−2.80)
11−12	15	62	0.242	0.62	56.26	130.18	2.10 (1.77−2.45)
12−13	16	47	0.340	0.44	38.01	73.92	1.57 (1.26−1.94)
13−14	18	31	0.581	0.53	22.60	35.91	1.16 (0.83−1.56)
14+	13	13	1	1.02	13.31	13.31	1.02 (0.49−1.60)

Table S27. Cohort life table of Jack Russell Terriers under primary veterinary care in the UK.

Age (year) [x, x+1)	Number of dogs died in [x, x+1) (d_x)	Number of dogs living at x (l_x)	Probability of dogs dying in [x, x+1) ($\hat{q}_x$)	Mean fraction of last year of life lived by dogs died in [x, x+1) ($\hat{a}_x$)	Number of dog−years lived in [x, x+1) (L_x)	Number of dogs living at year x (T_x)	Life expectancy at year x ($\hat{e}_x$)
0−1	14	1614	0.009	0.30	1604.19	20528.74	12.72 (12.53−12.90)
1−2	14	1600	0.009	0.50	1592.95	18924.55	11.83 (11.65−12.00)
2−3	17	1586	0.011	0.39	1575.55	17331.59	10.93 (10.75−11.1)
3−4	18	1569	0.011	0.54	1560.67	15756.04	10.04 (9.87−10.21)
4−5	18	1551	0.012	0.46	1541.33	14195.37	9.15 (8.99−9.31)
5−6	29	1533	0.019	0.51	1518.78	12654.05	8.25 (8.10−8.41)
6−7	33	1504	0.022	0.48	1486.69	11135.27	7.40 (7.26−7.55)
7−8	45	1471	0.031	0.53	1449.64	9648.57	6.56 (6.42−6.70)
8−9	55	1426	0.039	0.44	1395.25	8198.93	5.75 (5.62−5.88)
9−10	75	1371	0.055	0.47	1331.28	6803.68	4.96 (4.84−5.09)
10−11	119	1296	0.092	0.48	1234.13	5472.40	4.22 (4.11−4.34)
11−12	124	1177	0.105	0.53	1118.94	4238.27	3.6 0(3.49−3.71)
12−13	152	1053	0.144	0.48	973.58	3119.33	2.96 (2.86−3.07)
13−14	183	901	0.203	0.51	811.87	2145.75	2.38 (2.28−2.48)
14−15	219	718	0.305	0.46	599.58	1333.88	1.86 (1.76−1.96)
15−16	203	499	0.407	0.42	382.26	734.30	1.47 (1.37−1.58)
16−17	148	296	0.500	0.41	208.69	352.05	1.19 (1.08−1.31)
17−18	91	148	0.615	0.42	95.20	143.36	0.97 (0.83−1.11)
18−19	36	57	0.632	0.37	34.19	48.15	0.84 (0.65−1.05)
19+	21	21	1	0.66	13.96	13.96	0.66 (0.39−0.97)

Table S28. Cohort life table of Labrador Retrievers under primary veterinary care in the UK.

Age (year) [x, x+1)	Number of dogs died in [x, x+1) (d_x)	Number of dogs living at x (l_x)	Probability of dogs dying in [x, x+1) ($\hat{q}_x$)	Mean fraction of last year of life lived by dogs died in [x, x+1) ($\hat{a}_x$)	Number of dog−years lived in [x, x+1) (L_x)	Number of dogs living at year x (T_x)	Life expectancy at year x ($\hat{e}_x$)
0−1	17	2481	0.007	0.53	2473.00	29225.51	11.78 (11.67−11.89)
1−2	23	2464	0.009	0.46	2451.55	26752.51	10.86 (10.75−10.96)
2−3	16	2441	0.007	0.48	2432.65	24300.96	9.96 (9.86−10.06)
3−4	14	2425	0.006	0.47	2417.63	21868.31	9.02 (8.92−9.12)
4−5	22	2411	0.009	0.60	2402.21	19450.68	8.07 (7.98−8.16)
5−6	19	2389	0.008	0.49	2379.36	17048.47	7.14 (7.05−7.23)
6−7	44	2370	0.019	0.47	2346.52	14669.11	6.19 (6.10−6.28)
7−8	74	2326	0.032	0.59	2295.38	12322.59	5.30 (5.22−5.38)
8−9	111	2252	0.049	0.51	2198.11	10027.21	4.45 (4.38−4.53)
9−10	142	2141	0.066	0.49	2068.37	7829.10	3.66 (3.58−3.73)
10−11	245	1999	0.123	0.52	1882.15	5760.73	2.88 (2.81−2.95)
11−12	371	1754	0.212	0.48	1561.70	3878.58	2.21 (2.15−2.27)
12−13	441	1383	0.319	0.49	1157.54	2316.88	1.68 (1.62−1.73)
13−14	438	942	0.465	0.45	702.00	1159.34	1.23 (1.17−1.29)
14−15	314	504	0.623	0.42	322.71	457.34	0.91 (0.84−0.98)
15−16	137	190	0.721	0.40	107.35	134.63	0.71 (0.62−0.81)
16+	53	53	1	0.51	27.28	27.28	0.51 (0.34−0.71)

Table S29. Cohort life table of Pugs under primary veterinary care in the UK.

Age (year) [x, x+1)	Number of dogs died in [x, x+1) (d_x)	Number of dogs living at x (l_x)	Probability of dogs dying in [x, x+1) ($\hat{q}_x$)	Mean fraction of last year of life lived by dogs died in [x, x+1) ($\hat{a}_x$)	Number of dog−years lived in [x, x+1) (L_x)	Number of dogs living at year x (T_x)	Life expectancy at year x ($\hat{e}_x$)
0−1	19	196	0.097	0.37	184.11	1492.75	7.62 (6.99−8.20)
1−2	5	177	0.028	0.35	173.74	1308.65	7.39 (6.82−7.92)
2−3	15	172	0.087	0.29	161.40	1134.90	6.60 (6.06−7.14)
3−4	12	157	0.076	0.50	150.96	973.50	6.20 (5.70−6.69)
4−5	11	145	0.076	0.63	140.98	822.54	5.67 (5.20−6.12)
5−6	8	134	0.060	0.29	128.29	681.57	5.09 (4.65−5.50)
6−7	11	126	0.087	0.47	120.22	553.28	4.39 (3.99−4.79)
7−8	8	115	0.070	0.63	112.06	433.06	3.77 (3.40−4.14)
8−9	18	107	0.168	0.54	98.79	320.99	3.00 (2.64−3.37)
9−10	19	89	0.213	0.52	79.96	222.20	2.50 (2.15−2.85)
10−11	22	70	0.314	0.50	59.06	142.24	2.03 (1.69−2.41)
11−12	18	48	0.375	0.55	39.92	83.18	1.73 (1.37−2.12)
12−13	13	30	0.433	0.49	23.31	43.26	1.44 (1.06−1.85)
13+	17	17	1	1.17	19.96	19.96	1.17 (0.75−1.63)

Table S30. Cohort life table of Shih−tzus under primary veterinary care in the UK.

Age (year) [x, x+1)	Number of dogs died in [x, x+1) (d_x)	Number of dogs living at x (l_x)	Probability of dogs dying in [x, x+1) ($\hat{q}_x$)	Mean fraction of last year of life lived by dogs died in [x, x+1) ($\hat{a}_x$)	Number of dog−years lived in [x, x+1) (L_x)	Number of dogs living at year x (T_x)	Life expectancy at year x ($\hat{e}_x$)
0−1	21	635	0.033	0.40	622.30	7025.43	11.06 (10.73−11.40)
1−2	14	614	0.023	0.52	607.34	6403.13	10.43 (10.12−10.74)
2−3	15	600	0.025	0.54	593.09	5795.79	9.66 (9.36−9.95)
3−4	11	585	0.019	0.32	577.49	5202.69	8.89 (8.61−9.17)
4−5	20	574	0.035	0.50	563.92	4625.20	8.06 (7.79−8.32)
5−6	21	554	0.038	0.54	544.42	4061.28	7.33 (7.09−7.58)
6−7	13	533	0.024	0.45	525.85	3516.87	6.60 (6.37−6.83)
7−8	21	520	0.040	0.43	508.11	2991.02	5.75 (5.54−5.97)
8−9	31	499	0.062	0.45	481.95	2482.91	4.98 (4.77−5.18)
9−10	24	468	0.051	0.51	456.18	2000.96	4.28 (4.09−4.47)
10−11	53	444	0.119	0.50	417.61	1544.77	3.48 (3.30−3.66)
11−12	62	391	0.159	0.51	360.37	1127.17	2.88 (2.72−3.06)
12−13	74	329	0.225	0.48	290.80	766.80	2.33 (2.17−2.49)
13−14	79	255	0.310	0.51	216.18	476.00	1.87 (1.70−2.03)
14−15	68	176	0.386	0.48	140.65	259.82	1.48 (1.31−1.65)
15−16	62	108	0.574	0.43	72.38	119.18	1.10 (0.91−1.31)
16−17	25	46	0.543	0.34	29.40	46.79	1.02 (0.75−1.32)
17+	21	21	1	0.83	17.40	17.40	0.83 (0.48−1.26)

Table S31. Cohort life table of Springer Spaniels under primary veterinary care in the UK.

Age (year) [x, x+1)	Number of dogs died in [x, x+1) (d_x)	Number of dogs living at x (l_x)	Probability of dogs dying in [x, x+1) ($\hat{q}_x$)	Mean fraction of last year of life lived by dogs died in [x, x+1) ($\hat{a}_x$)	Number of dog−years lived in [x, x+1) (L_x)	Number of dogs living at year x (T_x)	Life expectancy at year x ($\hat{e}_x$)
0−1	5	785	0.006	0.52	782.60	9363.61	11.93 (11.69−12.13)
1−2	5	780	0.006	0.60	777.99	8581.01	11.00 (10.77−11.21)
2−3	13	775	0.017	0.47	768.11	7803.02	10.07 (9.85−10.28)
3−4	8	762	0.010	0.59	758.68	7034.91	9.23 (9.03−9.43)
4−5	9	754	0.012	0.43	748.84	6276.23	8.32 (8.13−8.51)
5−6	11	745	0.015	0.45	738.94	5527.40	7.42 (7.23−7.60)
6−7	18	734	0.025	0.39	723.01	4788.46	6.52 (6.35−6.70)
7−8	25	716	0.035	0.53	704.24	4065.44	5.68 (5.51−5.84)
8−9	34	691	0.049	0.60	677.29	3361.20	4.86 (4.71−5.02)
9−10	44	657	0.067	0.45	632.60	2683.91	4.09 (3.94−4.23)
10−11	63	613	0.103	0.44	577.82	2051.31	3.35 (3.21−3.48)
11−12	79	550	0.144	0.55	514.73	1473.49	2.68 (2.56−2.80)
12−13	103	471	0.219	0.50	419.35	958.75	2.04 (1.93−2.15)
13−14	147	368	0.399	0.48	291.37	539.40	1.47 (1.36−1.57)
14−15	106	221	0.480	0.45	163.17	248.04	1.12 (1.01−1.24)
15−16	86	115	0.748	0.40	63.80	84.86	0.74 (0.61−0.88)
16+	29	29	1	0.73	21.07	21.07	0.73 (0.47−1.03)

Table S32. Cohort life table of Staffordshire Bull Terriers under primary veterinary care in the UK.

Age (year) [x, x+1)	Number of dogs died in [x, x+1) (d_x)	Number of dogs living at x (l_x)	Probability of dogs dying in [x, x+1) ($\hat{q}_x$)	Mean fraction of last year of life lived by dogs died in [x, x+1) ($\hat{a}_x$)	Number of dog−years lived in [x, x+1) (L_x)	Number of dogs living at year x (T_x)	Life expectancy at year x ($\hat{e}_x$)
0−1	25	2347	0.011	0.51	2334.65	26599.22	11.33 (11.19−11.48)
1−2	28	2322	0.012	0.51	2308.21	24264.57	10.45 (10.31−10.59)
2−3	32	2294	0.014	0.57	2280.16	21956.36	9.57 (9.44−9.70)
3−4	38	2262	0.017	0.46	2241.59	19676.19	8.70 (8.57−8.83)
4−5	37	2224	0.017	0.48	2204.61	17434.60	7.84 (7.71−7.96)
5−6	52	2187	0.024	0.47	2159.42	15229.99	6.96 (6.85−7.08)
6−7	62	2135	0.029	0.45	2101.04	13070.56	6.12 (6.01−6.23)
7−8	102	2073	0.049	0.46	2018.34	10969.53	5.29 (5.18−5.40)
8−9	115	1971	0.058	0.46	1908.56	8951.18	4.54 (4.44−4.64)
9−10	169	1856	0.091	0.43	1760.36	7042.62	3.79 (3.70−3.89)
10−11	236	1687	0.140	0.48	1565.04	5282.26	3.13 (3.04−3.22)
11−12	262	1451	0.181	0.46	1310.79	3717.22	2.56 (2.48−2.65)
12−13	321	1189	0.270	0.47	1019.07	2406.43	2.02 (1.94−2.11)
13−14	329	868	0.379	0.49	698.62	1387.36	1.60 (1.52−1.68)
14−15	244	539	0.453	0.41	394.24	688.73	1.28 (1.19−1.37)
15−16	169	295	0.573	0.40	193.86	294.50	1.00 (0.89−1.11)
16−17	87	126	0.690	0.34	68.50	100.64	0.80 (0.66−0.95)
17−18	23	39	0.590	0.28	22.38	32.14	0.82 (0.58−1.09)
18+	16	16	1	0.61	9.76	9.76	0.61 (0.29−0.97)

Table S33. Cohort life table of Yorkshire Terriers under primary veterinary care in the UK.

Age (year) [x, x+1)	Number of dogs died in [x, x+1) (d_x)	Number of dogs living at x (l_x)	Probability of dogs dying in [x, x+1) ($\hat{q}_x$)	Mean fraction of last year of life lived by dogs died in [x, x+1) ($\hat{a}_x$)	Number of dog−years lived in [x, x+1) (L_x)	Number of dogs living at year x (T_x)	Life expectancy at year x ($\hat{e}_x$)
0−1	22	1039	0.021	0.30	1023.59	13026.19	12.54 (12.30−12.77)
1−2	8	1017	0.008	0.63	1014.03	12002.60	11.08 (11.59−12.01)
2−3	15	1009	0.015	0.44	1000.56	10988.56	10.89 (10.69−11.09)
3−4	8	994	0.008	0.59	990.75	9988.01	10.05 (9.86−10.24)
4−5	11	986	0.011	0.67	982.39	8997.26	9.13 (8.94−9.31)
5−6	19	975	0.019	0.37	962.99	8014.87	8.22 (8.04−8.40)
6−7	10	956	0.010	0.52	951.16	7051.87	7.38 (7.21−7.54)
7−8	18	946	0.019	0.48	936.69	6100.71	6.45 (6.29−6.61)
8−9	41	928	0.044	0.52	908.42	5164.02	5.56 (5.41−5.72)
9−10	51	887	0.057	0.55	864.04	4255.60	4.80 (4.65−4.95)
10−11	59	836	0.071	0.40	800.35	3391.56	4.06 (3.92−4.20)
11−12	94	777	0.121	0.54	733.83	2591.21	3.33 (3.21−3.47)
12−13	108	683	0.158	0.49	627.89	1857.38	2.72 (2.60−2.84)
13−14	149	575	0.259	0.52	503.96	1229.49	2.14 (2.02−2.26)
14−15	145	426	0.340	0.44	344.78	725.53	1.70 (1.58−1.82)
15−16	116	281	0.413	0.42	213.56	380.75	1.35 (1.23−1.48)
16−17	94	165	0.570	0.41	109.59	167.19	1.01 (0.88−1.15)
17−18	50	71	0.704	0.42	41.99	57.60	0.81 (0.64−0.99)
18+	21	21	1	0.74	15.61	15.61	0.74 (0.45−1.05)
